# Reaction-Driven Diffusiophoresis of Liquid Condensates: Potential Mechanisms for Intracellular Organization

**DOI:** 10.1021/acsnano.3c12842

**Published:** 2024-06-14

**Authors:** Gregor Häfner, Marcus Müller

**Affiliations:** † Georg-August Universität Göttingen, Institut für Theoretische Physik, Friedrich-Hund Platz 1, 37077 Göttingen, Germany; ‡ Max Planck School Matter to Life, Jahnstraße 29, 69120 Heidelberg, Germany

**Keywords:** diffusiophoresis, reaction cycles, theory and simulation, droplets, liquid condensates, kinetics of structure formation, self-organization

## Abstract

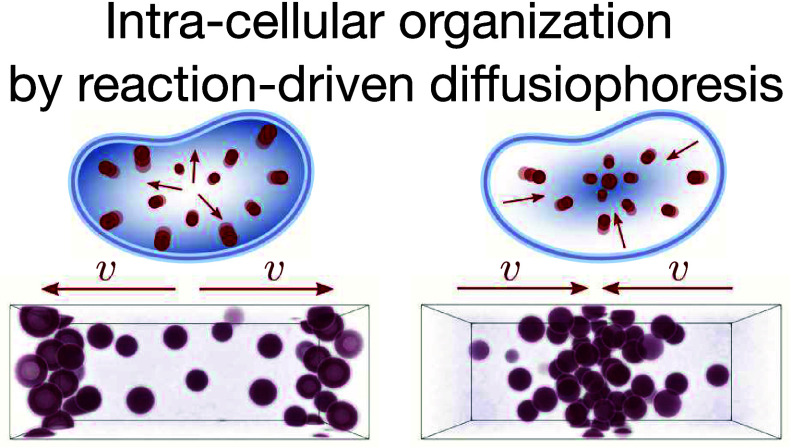

The cellular environment, characterized by its intricate composition and spatial organization, hosts a variety of organelles, ranging from membrane-bound ones to membraneless structures that are formed through liquid–liquid phase separation. Cells show precise control over the position of such condensates. We demonstrate that organelle movement in external concentration gradients, *diffusiophoresis*, is distinct from the one of colloids because fluxes can remain finite inside the liquid-phase droplets and movement of the latter arises from incompressibility. Within cellular domains diffusiophoresis naturally arises from biochemical reactions that are driven by a chemical fuel and produce waste. Simulations and analytical arguments within a minimal model of reaction-driven phase separation reveal that the directed movement stems from two contributions: Fuel and waste are refilled or extracted at the boundary, resulting in concentration gradients, which (i) induce product fluxes via incompressibility and (ii) result in an asymmetric forward reaction in the droplet’s surroundings (as well as asymmetric backward reaction inside the droplet), thereby shifting the droplet’s position. We show that the former contribution dominates and sets the direction of the movement, toward or away from fuel source and waste sink, depending on the product molecules’ affinity toward fuel and waste, respectively. The mechanism thus provides a simple means to organize condensates with different composition. Particle-based simulations and systems with more complex reaction cycles corroborate the robustness and universality of this mechanism.

The cellular environment, in which biochemical processes occur, is characterized by diverse local compositions with a high degree of complexity and spatial organization. There exists a multitude of organelles, which can be membrane-bound, such as mitochondria and lysosomes,^1^ and membrane-less, such as centrosomes, P granules or cajal bodies.^2−4^ The latter are typically formed by liquid–liquid phase separation.^5−8^ The spatial organization is integral to cells by fostering biochemical reaction cycles and orchestrating critical functions within cells, such as RNA metabolism and signaling.^9−12^ Understanding how these organelles are positioned and transported within the cellular milieu is a central question in cell biology.^13,14^ Typically, directed transport processes are thought of to rely on motor proteins or cytoplasmic flows.^14^ Since cells contain a strongly heterogeneous environment, transport processes due to concentration gradients have sparked interest, such as diffusiophoresis. This describes the movement of moieties in the concentration gradient of a different molecular species and has been thoroughly investigated in the context of hard colloidal particles.^15−19^ Diffusiophoresis was demonstrated to be responsible for directed transport of cargo in biological pattern forming systems^20,21^ and has been hypothesized to be implicated in the transport of intracellular condensates.^22^ However, biological condensates rarely are hard, impenetrable objects, but rather interact softly with their surrounding, including external diffusion and fluxes across interfaces and through the condensates. Since the effect was also observed for peptide and DNA condensates in salt concentration gradients,^23^ it is hence necessary to extend our understanding of motion induced by concentration gradients of a different species to liquid–liquid phase separation.

Reducing the complexity of biological systems experimentally and theoretically has proven a valuable tool to understand the underlying physics. Complex coacervates formed by charged polymers or proteins can mimic the fluid-like properties of membraneless organelles and can be engineered to involve or catalyze chemical reactions.^12,24−27^ Typically, their involvement in biochemical reactions keeps the organelles out of equilibrium, aiding to control their size and to suppress coalescence.^28,29^ Theoretically, these phenomena can be explained by the use of minimal models that feature phase separation in combination with simple reaction cycles. For instance, in a phase separating system, whose components can switch between hydrophilic and hydrophobic by driven reactions, coalescence arrests at a finite size of droplets, introducing a length scale.^30−32^ Chemical reaction cycles further have been demonstrated to influence nucleation dynamics^33,34^ or can even divide condensates.^35−38^ Catalytic properties for reactions of organelles in finite domains have also been shown to introduce positioning and self-propulsion.^39^

In this work, we demonstrate a fundamental mechanism to direct organelles to or away from the center of a finite domain, such as the cell lumen. We perform simulations with two complementary levels of description, a continuum model as well as a particle-based one. To understand the basic principles of diffusiophoresis of liquid phases, we start with a passive droplet (condensate) in externally maintained concentration gradients of a solute and a solvent. The latter has a strong repulsion to the droplet molecules, whereas the solute has varying interactions. Depending on the latter, there is a flux of the solute through the droplet that leads to its motion by virtue of the incompressibility of the solute, solvent, and the droplet liquid. We discuss the fundamental differences between the diffusiophoresis of liquid condensates and the one of colloids. Furthermore, we describe a minimal model, which involves a reaction cycle for molecules that dissolve in their precursor state, but phase separate from solution in their product state, termed reaction-driven assembly (RDA). To push the system out of equilibrium, the forward reaction is chemically driven, using a fuel to enable the reaction and creating waste in the process. Without exchange of chemical components with the surroundings, the system will convert all fuel and equilibrate into a homogeneous state. To avoid this and create a statistically stationary state out of equilibrium, fuel sources and waste sinks need to be introduced. In a biological context, these can arise from channels and activity at the cell membrane, whereas in synthetic systems, they can be introduced by a fluorinated oil phase that surrounds a microfluidic droplet, as recently demonstrated by Bergmann et al.^40^ The sinks and sources give rise to fluxes of fuel and waste. By accurately treating the incompressibility of the system, we demonstrate that this induces a product flux in opposite direction (directing the motion of condensates). The direction of transport crucially depends on the interaction of product with fuel and waste. Thus, this provides a simple means to grow and direct chemically fueled aggregates in a controlled manner. Analytically, we demonstrate that two additional, subdominant contributions to the movement arise from an asymmetric production in the condensates’ surroundings as well as an asymmetric reversion of product on the inside. Finally, we illustrate the robustness of the results for a selection of scenarios: Combining passive and RDA-formed droplets, we show that the mechanism allows to simultaneously move droplets in opposite direction. Additionally, we consider a system, where product is amphiphilic and self-assembles into micelles or vesicles. Here the favorable interactions of the hydrophilic head groups with fuel suffice to guide their growth and direct the aggregates’ motion.

## Results and Discussion

We consider a solution, consisting to a majority of solvent *S*, in which components undergo a simple reaction cycle. In a forward reaction, a hydrophilic reactant *R*, acting as a precursor, reacts with a fuel *F*, a high-energy molecule. The reaction creates a hydrophobic product *P*, that phase separates from solution, and some waste *W*, *i. e.*, *R* + *F* → *P* + *W* with forward reaction rate constant *r*_*f*_. The reaction cycle is completed by a spontaneous reversion of the product molecule to precursor, *P* → *R*, at reaction rate constant *r*_*b*_.

We perform continuum model simulations where the system state is fully characterized by the normalized segmental densities which we refer to as concentrations in this work, ϕ_*c*_(**r**) = ϱ_*c*_(**r**)*V*/*∑*_*c*_*n*_*c*_*N*_*c*_ for *c* = *P*, *R*, *S*, *F*, *W*, with the number of molecules, *n*_*c*_, the molecular discretizations, *N*_*c*_, and the segment number densities, ϱ_*c*_(**r**). The segmental volumes do not change and are identical for all species. Thus, local volume fractions and local concentrations, ϕ_*c*_(**r**), are identical.

The system’s equilibrium is governed by the Flory–Huggins-de Gennes free-energy functional,^41^
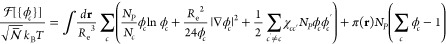
1

The mean end-to-end distance, *R*_e_, of the product molecule sets the length scale, and we measure all energies in units of *k*_B_*T*, with *k*_B_ being the Boltzmann constant and *T* the temperature, respectively. *N*_*P*_ = *N*_*R*_ denote the chain discretization (proportional to molecular volume) of the product or reactant polymer, and the invariant degree of polymerization, , dictates the strength of thermal fluctuations.^42,43^*N*_*c*_ with *c* = *S*, *F*, and *W* denote the discretization of solvent, fuel, and waste. The binary interactions are determined by the Flory–Huggins parameter, χ_*cc*′_, and the system is considered incompressible, demanding *∑*_*c*_ϕ_*c*_ = 1, which is enforced by the Lagrange multiplier, π(**r**).

The dynamics of the concentration fields account for diffusive equilibration and driven, chemical reactions,
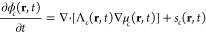
2

The first term minimizes the free energy, locally conserving the concentration. Here gradients of the chemical potentials, , give rise to volume-fraction fluxes, **j**_*c*_ = −Λ_*c*_∇μ_*c*_. The Onsager coefficient Λ_*c*_ is proportional to the diffusion coefficient, *D* = *λR*_e_^2^ which defines a time scale . Thermal noise is included as noise in the flux, see eq 9. The second term in the time-evolution equation, *s*_*c*_(**r**), describes the chemical reactions, which are considered to follow mass-action kinetics. The model and parameters are described in more detail in the Sec. Methods.

Although the parameters of the Flory–Huggins-de Gennes free-energy functional are inspired by polymer physics, the free-energy functional and the kinetic equations describe the universal features of macrophase separation coupled to reactions and, thus, are applicable to a wide variety of systems.^37,44^ In this work we do not consider effects that arise from hydrodynamics, which could be incorporated using multifluid continuum models,^45^ or multiparticle collision dynamics.^46,47^ This is valid for the slow transport of droplets in a highly viscous environment. Notably diffusiophoretic movement of liquid condensates can also occur in this limit.

To illustrate the relevant effects, we consider four scenarios with increasing complexity: (i) We start with a phase-separating but chemically inactive system subjected to an externally maintained flux of solute and solvent. (ii) We continue with chemically active systems, where droplets form by RDA, and the continuous turnover of fuel to waste and the concomitant local refilling of the former naturally gives rise to concentration gradients. Here, either waste or fuel are only implicitly represented and treated to be part of the solvent. (iii) Then, both components are treated explicitly, and (iv) we show that two distinct condensate types with different interactions of their constituents can move in opposite direction and (v) we show an exemplary scenario where the product is amphiphilic and assembles into micelles or vesicles that are also transported.

### Passive Droplets in External Concentration Gradients

To introduce the basic concept of diffusiophoresis for phase-separated condensates, let us begin with passive droplets in externally maintained concentration gradients but without chemical reactions. For this system, we only require three components, the droplet material, *P*, a solvent, *S*, and the solute, *F*. The solvent is highly repulsive toward the droplet material χ_*PS*_*N*_*P*_ = 30, giving rise to liquid–liquid phase separation, whereas we vary the interactions of the solute with the droplet material, χ_*PF*_*N*_*P*_ = −30, 0, and 30. In the remainder of this work, *F* will take the role of a chemical fuel that enables the forward reaction, but it is purely passive in this section.

A concentration gradient is maintained by introducing local sources and sinks. At the sides of the domain *z* = 0, we place an *F*-source and *S*-sink, *S* → *F*, while in the center slab, *z* = *L*_*z*_/2 = 12.8*R*_e_, the opposite happens, *F* → *S*, with higher rate constant. Starting without droplet material in the system, stationary concentration profiles build up with ϕ_*F*_(*z* = 0) ≈ 0.1 and approximately linearly decreasing toward the center, ϕ(*z* = *L*_*z*_/2) ≈ 0. In this steady state, there is a continuous flux of solute from the side, *z* = 0, into the center, *z* = *L*_*z*_/2, and a flux of solvent in opposite direction. Next, we replace the solvent by droplet material inside a sphere of radius *R* ≈ 1.4*R*_*e*_ slightly off centered in the domain. The droplet rapidly equilibrates locally and interfaces build up. Since the solvent is strongly repelled from the droplet phase, its flux inside almost vanishes. The solute on the other hand, may have nonpreferential or attractive interactions toward the droplet material. In these scenarios, it has a finite concentration inside the droplet and diffuses across its interfaces and through it. Incompressibility demands the total concentration be constant and with the assumption of high viscosity the total flux vanishes,
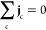
3

This is also the case with reactions in the later sections, because, in our case, these conserve volume. This condition couples the finite solute flux, **j**_*F*_, inside the droplet to the flux of droplet material, **j**_*P*_. Outside the droplet, ϕ_*P*_ ≈ 0, we have **j**_*F*_ ≈ – **j**_*S*_, whereas inside the droplet, ϕ_*S*_ ≈ 0, the flux of solute cancels the flux of droplet material, **j**_*P*_ ≈ – **j**_*F*_. Thus, the passive droplet moves in the opposite direction than the *P*-attractive solute, *i. e.*, toward the side of the simulation cell.

**Figure 1:**
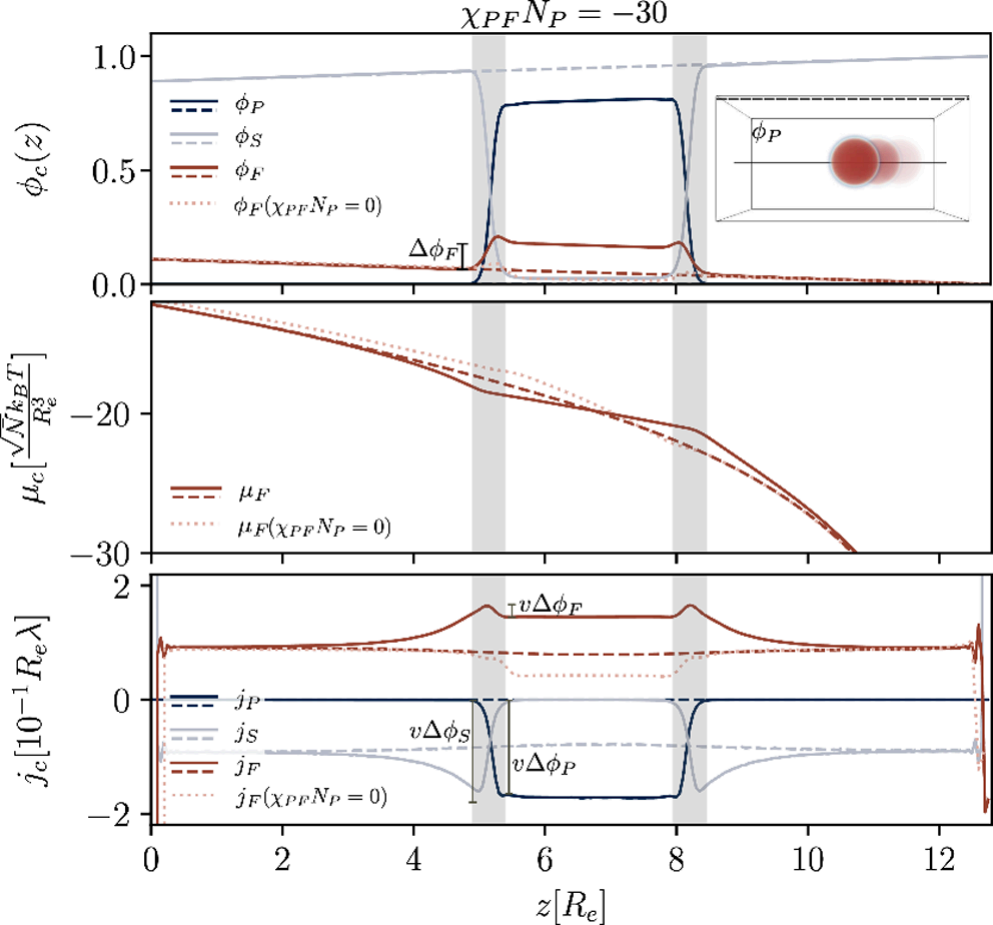
Exemplary profiles, 0 ≤ *z* ≤ *L*_*z*_/2, of a passive droplet in external concentration gradients for preferential interactions with the solute, χ_*PF*_*N*_*P*_ = −30, at time *tλ* = 20. Concentration profiles (top), chemical potential of the solute (middle) and fluxes (bottom) plotted along two lines, one that passes by the droplet (dashed) and one that runs through its center (solid). In the bottom panel, the flux jumps, *v*Δϕ_*c*_ with *v* = −0.21*R*_e_λ, are indicated for each component. Example snapshots of the 3D-morphology are overlaid in the inset of the top panel, and the positions of the line profiles–inside and outside of the droplet–are indicated. Additionally, each panel shows the respective quantity for the solute for the neutral interaction, χ_*PF*_*N*_*P*_ = 0 at time *tλ* = 62, as dotted pink lines. Notice that the reference (far-field, outside the droplet) chemical potential is slightly shifted compared to that of the system with favorable interactions, but not shown here.

Figure 1 shows example profiles for the attractive scenario, χ_*PF*_*N*_*P*_ = −30, giving concentrations, solute chemical potential and fluxes for a cut through the system that crosses (solid) and excludes (dashed) the droplet, respectively. Clearly, for the attractive scenario, solute is enriched inside the droplet. The interfaces are *locally* in equilibrium, *i. e.*, the chemical potentials are continuous across the narrow interface, as shown exemplary for μ_*F*_. Under the assumption of a sharp interface, its movement in *z*-direction demands a discontinuity of the *F*-flux, Δ*j*_*F*,*z*_ = *v*Δϕ_*F*_, which is indicated in the figure, where *v* denotes the velocity of the droplet. Likewise, there must be an analog interfacial discontinuity, Δ*j*_*P*,*z*_ = *v*Δϕ_*P*_, in the *P*-flux and in the *S*-flux. In order to fulfill the requirement, Δ*j*_*F*,*z*_/Δϕ_*F*_ = Δ*j*_*P*,*z*_/Δϕ_*P*_ = Δ*j*_*S*,*z*_/Δϕ_*S*_ at the interface, the concentration profiles around the droplet’s interface are altered, resulting in a smaller chemical potential gradient of *F* inside of the droplet and a larger one in front of the interface, outside of the droplet, see middle panel of Figure 1. The concomitant fluxes along the *z*-direction are depicted in the bottom panel. By virtue of incompressibility, *j*_*P*,*z*_ ≈ – *j*_*F*,*z*_ and *j*_*S*,*z*_ ≈ 0 inside of the droplet. The panel also indicates the three interfacial jump conditions, Δ*j*_*c*,*z*_ = *v*Δϕ_*c*_ for *c* = *P*, *F*, and *S*, at the droplet’s interface which are blurred by the finite interface width.

**Figure 2:**
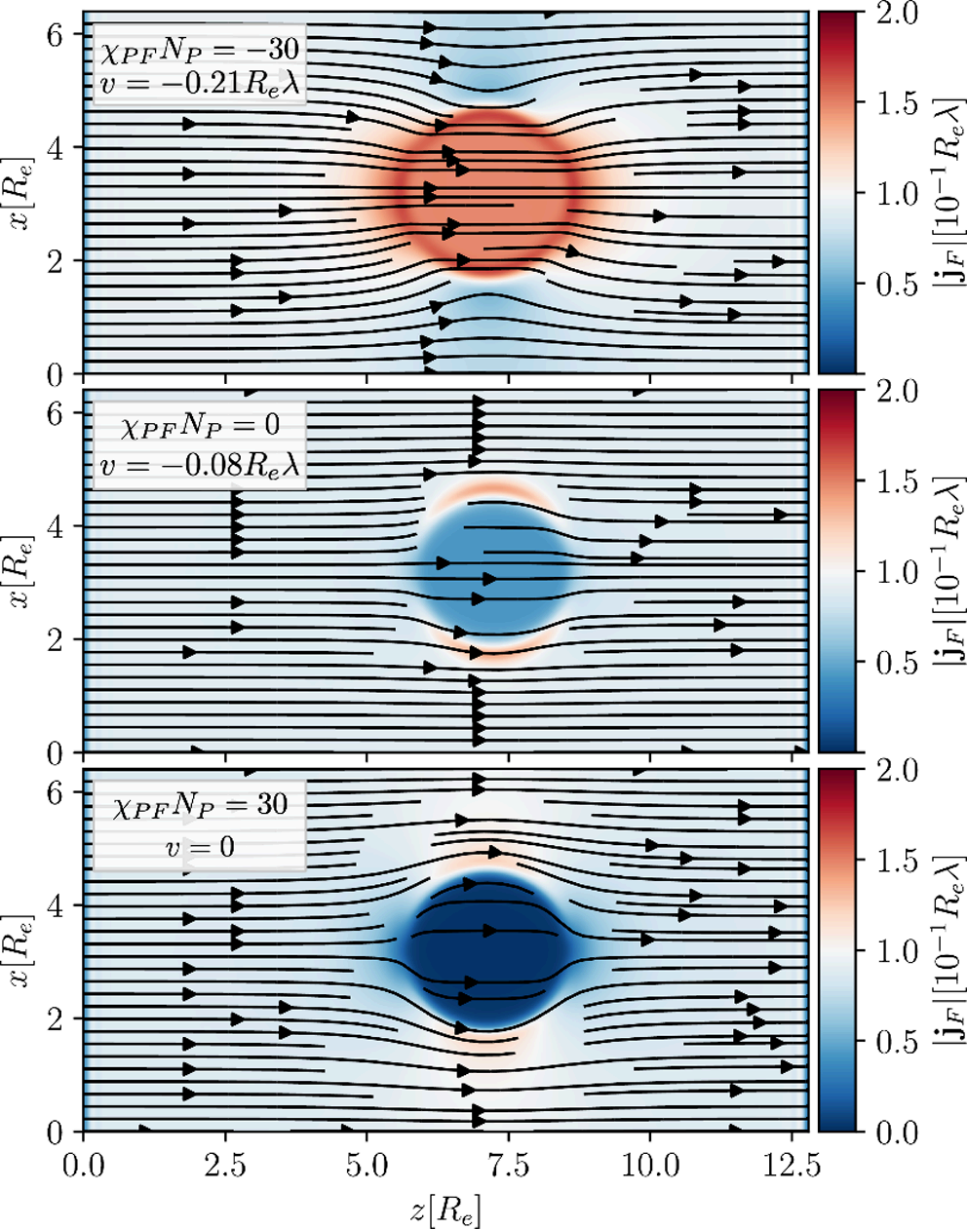
Solute fluxes, plotting the direction as arrows and the absolute values colored underneath, for the different interaction scenarios: (top) χ_*PF*_*N*_*P*_ = −30 at *tλ* = 20, (middle) χ_*PF*_*N*_*P*_ = 0 at *tλ* = 62, (bottom) χ_*PF*_*N*_*P*_ = 30 in the stationary state, where the droplet remains at the position it was set up on. Shown is a slice through the center of the *y*-dimension.

The one-dimensional flux profiles in Figure 1 only provide an incomplete description (also see Sec. 1D Passive Droplets in External Concentration Gradients of the Supporting Information (SI)), and the cylinder-symmetrical solute flux around the droplet is illustrated in Figure 2. For an attraction, χ_*PF*_*N*_*P*_ = −30, between solute and droplet material, solute preferentially diffuses through the droplet and the flux is focused into the droplet. This, in turn, increases the *P*-flux in opposite direction, *j*_*P*,*z*_ ≈ – *j*_*F*,*z*_, and quickly moves the droplet toward the *F*-source, at a velocity *v* = −0.21*R*_e_λ.

In the opposite case, if there is a strong repulsion toward the droplet material, χ_*PF*_*N*_*P*_ = χ_*PS*_*N*_*P*_ = 30, the concentration of solute inside of the droplet is vanishingly small, just as the solvent concentration is. Both, solvent and solute preferentially avoid the droplet and flow past it. In this case, the droplet does not move at all, *v* = 0.

In the intermediate case of nonpreferential interactions, χ_*PF*_*N*_*P*_ = 0, our choice of a higher molecular volume of *P*, *N*_*P*_ = 10*N*_*F*_ leads to an entropy-induced reduction of solute concentration inside of the droplet compared to the immediate outside (as indicated by the dotted line in the upper panel of Figure 1). This leads to a deflection of the *F*-flux around the droplet. The flux inside the droplet is reduced and, therefore, also the droplet velocity is smaller compared to the *P*-*F*-attractive scenario, *v* = −0.08*R*_e_λ. The solute concentration, chemical potential, and flux are plotted as dotted lines in Figure 1 for comparison.

The measured droplet velocity can be related to fluxes and concentrations, as worked out in Sec. Theoretical Prediction of Velocities of the SI for the general RDA case. In the limit of no reactions and small variation of concentration profiles across the droplet volume, the comparison reduces to . This comparison is demonstrated in Sec. Velocity of Passive Droplets in External Concentration Gradients of the SI.

Summarizing this section, external concentration gradients can move passive, phase-separated droplets by diffusive solute fluxes across the interfaces and through these droplets. The flux of droplet material couples to the solute flux via incompressibility. In this way the droplet is transported toward the solute source. The droplet velocity is the higher, the more enriched the solute is inside of the droplet. This concept will reappear in the chemically active systems with RDA-formed droplets in the next sections.

### Diffusiophoretic Velocity from Metabolism

Estimating the significance of the effect in living cells, we take the metabolite ATP as an example. Its molecular concentration gradient, as estimated by Sear in ref (22), can become roughly |∇ρ_ATP_|∼ 10^5^ – 10^6^/μm^–4^ for molecular concentrations on the order of ρ_ATP_ ∼ 10^7^μm^–3^. Taking a molecular volume of 1/ρ_0_ ∼ 10^–9^μm^3^ results in a normalized volume concentration of ϕ_ATP_ = ρ_ATP_/ρ_0_ = 10^–2^ and a gradient of |∇ϕ_ATP_| = |∇ρ_ATP_/ρ_0_|∼ 10^–4^ – 10^–3^μm^–1^. With the diffusion coefficient of *D*_ATP_ ∼ 10^2^μm^2^/s^48^ this results in an ATP flux of *v* ∼ *j*_ATP_ ≈ *D*_ATP_|∇ϕ_ATP_|∼ 10–100 nm/*s* inside the solution. Hence, this is the order of magnitude, one can expect for the transport velocity at neutral interactions, independent of condensate size. This constitutes just one component, but in cells concentration gradients can be present in an abundance of components. With attractive interactions, the transport efficiency can even be increased.

Let us recall the mechanism for diffusiophoresis of colloid particles.^15−19^ In this case, an osmotic pressure gradient in a thin surface layer gives rise to a slip velocity, i.e. a relative motion of colloid and fluid. In order to move the colloid, a hydrodynamic mass flux is required, whereas in the case of liquid condensates, a diffusiophoretic movement can be observed even at high dynamic viscosity, μ̃, i.e. in the absence of a total flux. Importantly, prior theories of diffusiophoresis of liquid condensates^17,49^ ignored the multicomponent nature of phase separation and diffusion across interfaces and through the liquid phases, treating the phenomenon on the same footing as for colloids, which is valid only for strongly segregated condensates. In this case, interactions that cause the movement only occur at a thin surface layer of width, *b*, and the colloidal diffusiophoretic velocity scales like .^16,18^ The ratio of the velocities thus scales like , and the two mechanisms of diffusiophoresis depend on different control parameters. Experimentally, both effects are simultaneously present and a hydrodynamic treatment that enables to account for both effects (i.e., model-H dynamics instead of model-B dynamics^50^) would be valuable.

### RDA-Formed Droplets with Implicit Waste or Fuel

In the following, we consider reactions, where the droplet material, product *P*, is continuously turned over into a precursor state, reactant *R*, that is hydrophilic and expelled from the droplet. The precursor, *R*, can be transformed into the product, *P* by a reaction with fuel, *F*, which is converted to waste, *W*, in the reaction. Hence, the whole system acts as a fuel sink and a waste source. In a first step, we treat one of the latter components implicitly, as part of the solvent. In the following, we treat the systems with thermal noise, since these systems rely on frequent nucleation of condensates.^51^

#### Implicit Waste

When treating waste implicitly, we only consider the four components *P*, *R*, *S*, and *F*, and lump the waste together with the solvent. Hence, the forward reaction becomes *R* + *F* → *P* + *S*, and the fuel source simultaneously acts as the waste (solvent) sink.

**Figure 3:**
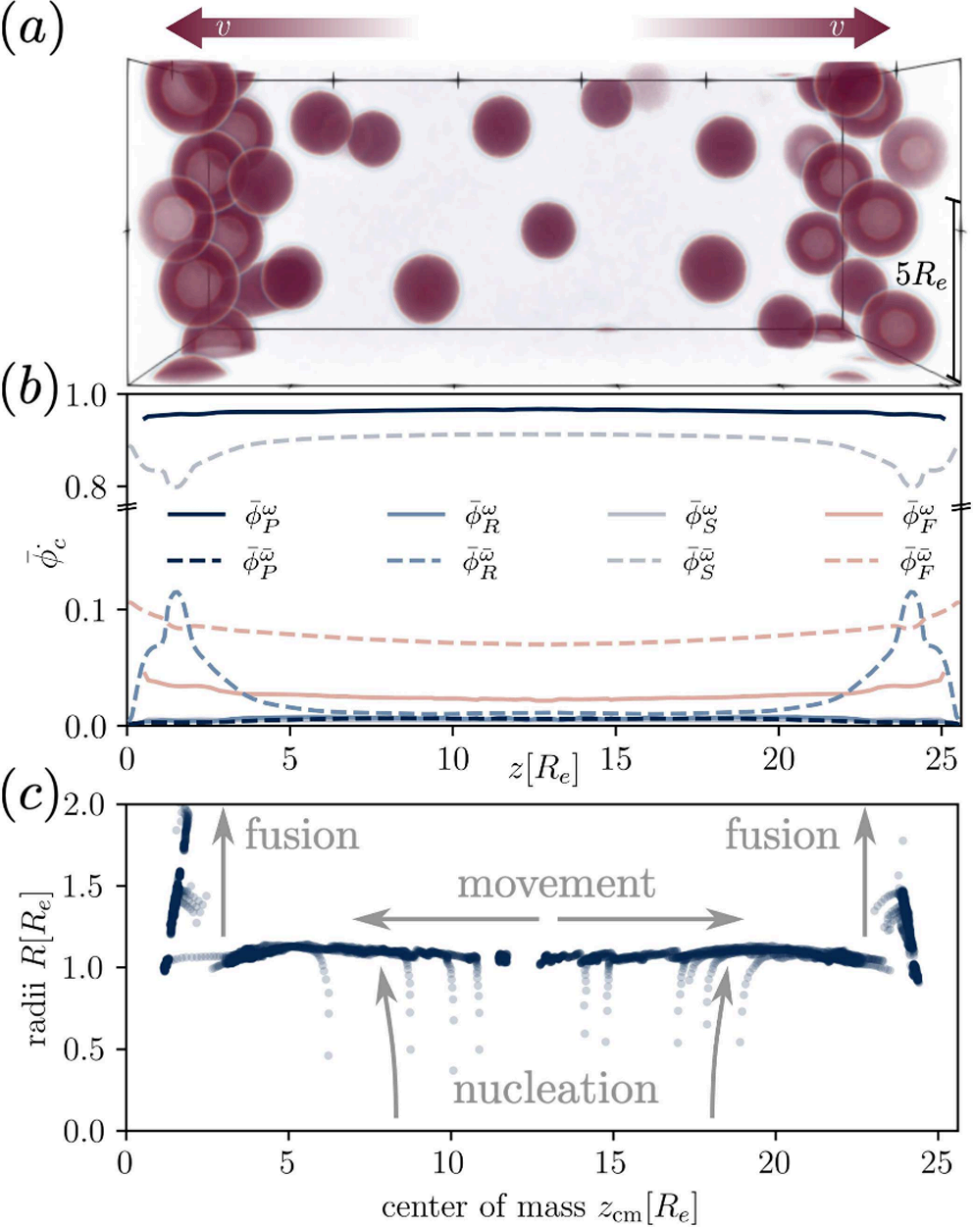
Simulation results for implicit waste treatment for reaction rate constant, *r*_*b*_ = 0.08λ. The fuel is nonpreferential toward the product. (a) Example morphology at time *tλ* = 400 showing the local product concentration ϕ_*P*_(**r**). The movement of droplets is indicated by the arrows. (b) Laterally averaged concentration profiles inside,  (solid), and outside of the droplets,  (dashed), as a function of *z*. (c) Individual droplet radii plotted against their center of mass, *z*_cm_. The droplet dynamics that determine the shape of the plot are indicated.

For a backward reaction rate constant of *r*_*b*_ = 0.08λ and nonpreferential interaction of fuel and product, χ_*PF*_*N*_*P*_ = 0, an example morphology is shown in Figure 3 (a), and the full dynamics of the concentration field can be appreciated in Supplementary Movie I. Upon start of the simulation, the source injects fuel, *F*, at the sides of the simulation cell and *F* diffuses into the bulk where the forward reaction commences with precursor molecules, *R*. Since fuel is continuously used within the bulk but only at the sides of the domain, an inhomogeneous but stationary concentration profile builds up quickly, with highest concentration at the source, *i. e.*, ϕ_*F*_ decreases toward the center of the simulation cell. This implies a continuous flux of fuel toward the center. By virtue of incompressibility, waste, which is part of the solvent and produced in the bulk, flows continuously back. The forward reaction creates product whose concentration quickly surpasses the binodal at ϕ_*P*,binodal_ ≈ 0.002 and approaches the mean-field spinodal, ϕ_*P*,spinodal_ ≈ 0.01, and phase separates from the solution. The droplet-creation and growth process is illustrated in Sec. Nucleation of RDA-formed Droplets of the SI. The coarsening, however, quickly arrests at a finite droplet size that is dictated by the continuous reactions. The hydrophilic precursor has a finite lifetime and thus a finite diffusion length in the solution, and the same holds true for the product in the droplets. Hence, the system locally displays the same behavior as systems with homogeneous fuel concentration.^30,32,52^ As before, the nonpreferential interactions between product and fuel lead to a finite fuel concentration inside the droplets. Thus, droplets are transported toward the fuel source, *z* = 0 or *L*, leaving larger space of solvent phase between condensates at the center of the simulation cell, where the commencement of the forward reaction, again, leads to an oversaturation of product in the solvent phase, which nucleates new (trailing) droplets.

Arriving at the sides of the simulation cell, the droplet motion stalls (due to a wall that prevents droplets from screening the fuel source). Here, the droplets fuse with trailing ones, become supersized (*i. e.*, larger than dictated by the reactions), and immediately shrink again. This fusion-shrinking cycle establishes a statistically stationary state at the boundaries of the simulation cell.

Using a Hoshen-Kopelman cluster analysis (HKCA),^53,54^ we determine the droplet domains, ω_*i*_, where *i* indexes the droplets and use these to temporally and locally average the concentration profiles inside and outside of the droplet. This is shown in Figure 3 (b). Visibly droplets consist of product and fuel only, as the precursor and solvent concentrations practically vanish inside.

The solvent phase chiefly consists of solvent, fuel, and precursor. The product density is small in the solvent phase because of the rather large incompatibility between *P* and *S*, and the resulting small binodal concentration. Nonetheless, the product concentration can locally and temporarily become larger than the averaged concentration when the movement of droplets creates a void space between the droplets at the center of the simulation cell. Subsequently, this gives rise to the creation of a new droplet.

As the droplets move toward the sides, the product decays into precursor, *R*, and the precursor concentration is large in the solvent phase near the source. Thus, there is a backward flux of precursor from the sides into the center of the simulation cell that allows to create droplets at the center. In summary, the reaction cycle gives rise to two distinct flux cycles: (i) Fuel is transported from the source into the center, whereas waste flows the opposite way. The (partial) fuel fluxes through the droplets results in a transport of droplets toward the fuel source. (ii) Since product continuously reverts to precursor (reactant), precursor has a high concentration where product droplets move to, *i. e.*, the fuel source. From here precursor flows in opposite direction toward the center.

The above-described life of droplets - creation, movement, fusion and shrinkage - can also be appreciated when using the HKCA to plot the droplet radii, *R*, determined as in ref (52), against their center of mass, *z*_cm_. This is shown in Figure 3 (c). Upon nucleation the droplets quickly approach a uniform size, which varies only slightly over the course of the movement. Near the source, however, fusion of droplets leads to droplet sizes that are far larger than the reaction-dictated one. In extreme cases, the turnover of product on the inside produces more precursor than can diffuse to the outside solution. This leads to an enrichment of precursor, which nucleates a hydrophilic core. This way, a spherical shell emerges. Such vacuolization has recently been shown, both experimentally and theoretically, to be stable in the presence of the here-investigated reaction cycles.^40,55^ In our case, this happens after rapid fusion, which can also appear at the center of the domain in other scenarios and is not unique to the specific boundary condition. The large moieties at the side of the domain, spheres and shells are closely packed, because they are stopped by the wall, while trailing condensates still move. This implies a high competition for product among these, and an imbalance of product influx into the condensate and decay of product on the inside, resulting in shrinkage.

To show that our choice of boundary conditions is irrelevant for the overall effects in the system we performed simulations without the wall, using fully periodic boundary conditions. This leads to fusion of oncoming droplets immediately at the source, *z* = 0 aka *z* = *L*. One also observes the same movement and vacuolization, which is demonstrated in Sec. Choice of Boundary Conditions of the SI. In addition, we demonstrate that a larger simulation-cell size can lead to nucleation of small droplets that slowly grow upon approaching the fuel source.

**Figure 4:**
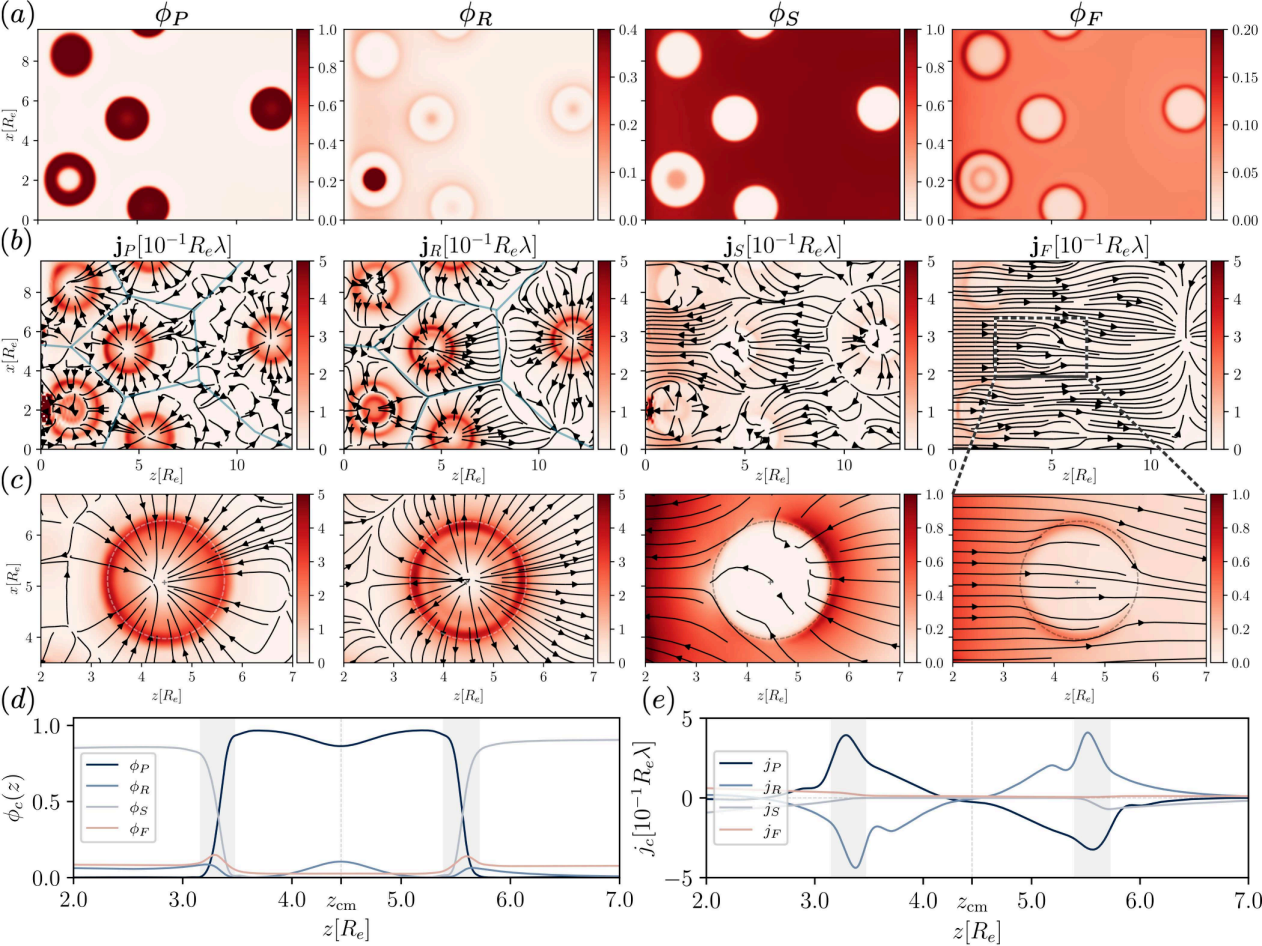
Example concentration fields and fluxes for the simulation shown in Figure 3 at time *tλ* = 870 for a slice through the domain, showing the *x*-*z*-plane at *y* = 1.8*R*_e_. (a) Concentration fields, ϕ_*c*_. (b) Corresponding flux fields, **j**_*c*_, showing the direction as arrows and the magnitude |**j**_*c*_| as the color indicated by the color bars. The Voronoi tessellation is indicated in turquoise. (c) Flux fields, zoomed into a single droplet, as indicated for **j**_*F*_. The center of mass and interface position are indicated. (d) Concentration profiles for a line at *x*_cm_ = 5.05*R*_e_ and (e) corresponding fluxes . In the latter two panels, the droplet center and the interface region are indicated, as dashed vertical line and shaded region, respectively.

The directed movement of droplets participating in the reaction cycle is also rooted in the presence of concentration gradients, *i. e.*, *reaction-driven diffusiophoresis*. It is similar to the case of passive droplets but is additionally influenced by the reactions. Since the fluxes in this system can become rather complex, let us have a detailed look at these for a representative snapshot, plotting a two-dimensional (2D) slice through the *x*-*z*-plane of the 3D domain. Figure 4 shows the concentration fields, the resulting fluxes, and a zoom-in on a single droplet in an example morphology (2D slices and corresponding profiles through the center of the droplets). For this, we filtered the thermal noise by running the simulation without it for a short time, *tλ* = 0.1, as described in Sec. Analysis Procedure in the SI.

We can see that the droplets consist majorly of product, with a small fuel concentration remaining, which is slightly enriched at the interface to screen the interactions of hydrophobic product and solvent. The precursor concentration, *R*, is high at the center of the droplet and decreases close to the interface toward an equilibrium concentration. In the case of a spherical shell the precursor is strongly enriched on the inside, while the opposite is true for fuel and solvent.

From the flux plots we can see that there is a continuous eflux of precursor, *R* out of the droplets and a concomitant influx of product. It is largest close to the interface. This is the result of the driven chemical reactions: On the one hand, the backward reaction produces hydrophilic precursor in the hydrophobic environment of a droplet, which diffuses out of the droplet. On the other hand, the forward reaction creates hydrophobic product in the solvent phase by conversion of the high-energy fuel molecules. This leads to a product flux toward the droplets on the outside. The behavior is studied in detail in ref (35).

In our scenario, droplets are usually densely packed and therefore compete for product. In Figure 4 (b) we can observe that product fluxes always point into the direction of the nearest RDA-formed droplet. Using a Voronoi tessellation,^56,57^ we assign a region surrounding each droplet as the accumulation volume. These Voronoi domains, Ω_*i*_, with *i* indexing the droplets are delineated by a turquoise line in the plot. The morphology of these domains is analyzed in detail in Sec. Reaction Contribution to Velocities in the SI, for representative scenarios, and we discuss their influence on droplet movement.

From the close-ups of the flux fields we can see that the solvent flux almost vanishes inside the droplet, whereas the fuel, though slightly deflected, flows through it. This is similar to the case of passive droplets in an external gradient. Product and precursor gain additional radial fluxes, toward and away from the center of the droplet, respectively. While the precursor flux appears mirror symmetric with respect to the droplet’s center, the product flux is slightly off-centered. This can also be appreciated in the profile of Figure 4 (e): The product flux is slightly smaller than zero in the droplet center, whereas the fuel flux is slightly positive inside the droplet.

From Figure 4, we can qualitatively identify three contributions that lead to movement of the RDA-formed droplets: (i) The finite fuel flux inside the droplets demands a product flux in opposite direction. In principle, the same applies for the waste (aka solvent) flux, which in this scenario is negligible inside the droplet. (ii) The production inside the accumulation volume creates product on the outside of the droplet, which leads to an influx of product into the droplet. Asymmetry in production can thus lead to motion, if the influx is higher on one side. Such asymmetry can arise both, from concentration gradients of fuel and precursor or an asymmetric placement of the droplet in its accumulation volume, respectively. For this argument, we assume the droplets are exclusively supplied by product generated in its Voronoi cell (aka the accumulation domain), Ω_*i*_. In the absence of concentration gradients, a droplet adopts a finite size, for which the loss of droplet material (product), which reverts into precursor inside the droplet, matches the gain due to the forward reaction, *R* + *F* → *P* + *S*, in its Voronoi cell. In our scenario, precursor and fuel have a higher concentration closer to the fuel source, resulting in an imbalance in production that provides an additional cause of directed droplet motion toward the fuel source. (iii) The backward reaction may also lead to asymmetric efflux of precursor, which would shift the droplet’s positions.

In Sec. Theoretical Prediction of Velocities of the SI, we analytically calculate the velocity of RDA-formed droplets, thereby quantifying the above-mentioned contributions. To this end, we use the approximations that (i) interfaces are sharp, (ii) product material that is generated inside the Voronoi cell, Ω_*i*_ of the individual droplet, *i*, instantly condensates on the surface of the droplets, and (iii) product that decays inside the droplet is instantly refilled from the interface. The latter two approximations, the *instantaneous condensation approximations* (ICA), essentially replace the fluxes that arise from forward and backward reaction of product in Figure 4 by sources and sinks immediately at the droplet interface and retain only the (nonreactive) fluxes that arise from fuel and waste through incompressibility, *i. e.*, passive diffusiophoresis of liquid condensates, as discussed in Sec. Passive Droplets in External Concentration Gradients. The approximation is valid if the concentration fields change only slowly during the diffusion time of a single molecule across the accumulation domain.

With these approximations we can now identify the (nonreactive) product flux inside the *i*^th^ droplet, **r** ∈ ω_*i*_, as in the case of passive diffusiophoresis, **j**_*P*_^nr^(**r**)=-[**j**_*F*_(**r**)+**j**_*W*_(**r**)]. We obtain for the droplet’s velocity, *v*_*i*_ by
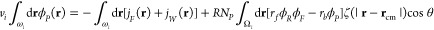
4where  are the longitudinal components of the fluxes. The second term corresponds approximately to the precursor contribution of the product flux, where the factor ζ(|**r** – **r**_cm_|) cos θ, with θ being the angle of **r** – **r**_cm_ to the transport direction, , accounts for both, the finite lifetime of the product during which it reaches the droplet surface and the location on the droplet surface, at which the product attaches after it has been generated inside the Voronoi cell. This term arises from the instant placement of the net reaction product in the accumulation domain onto the surface, weighted by ζ(|**r** – **r**_cm_|), instead of directly treating the precursor flux.

eq 4 identifies the three different contributions to the droplet’s velocity: (i) the first integral on the r.h.s. denoted as *nonreactive flux contribution*, (ii) the *forward reaction contribution*, corresponding to the first summand in the second integral, and (iii) the *backward reaction contribution*, represented by the second term of the second integral. While the first contribution represents the mechanism of the above case of passive diffusiophoresis, the latter two correspond to an asymmetric in- and efflux at the surface of the droplet. Hence, the motion also results from growth in the front and shrinkage in the back of the droplet rather than transport, corresponding to existing theory of droplet ripening in regulator concentration gradients.^37,58^

**Figure 5:**
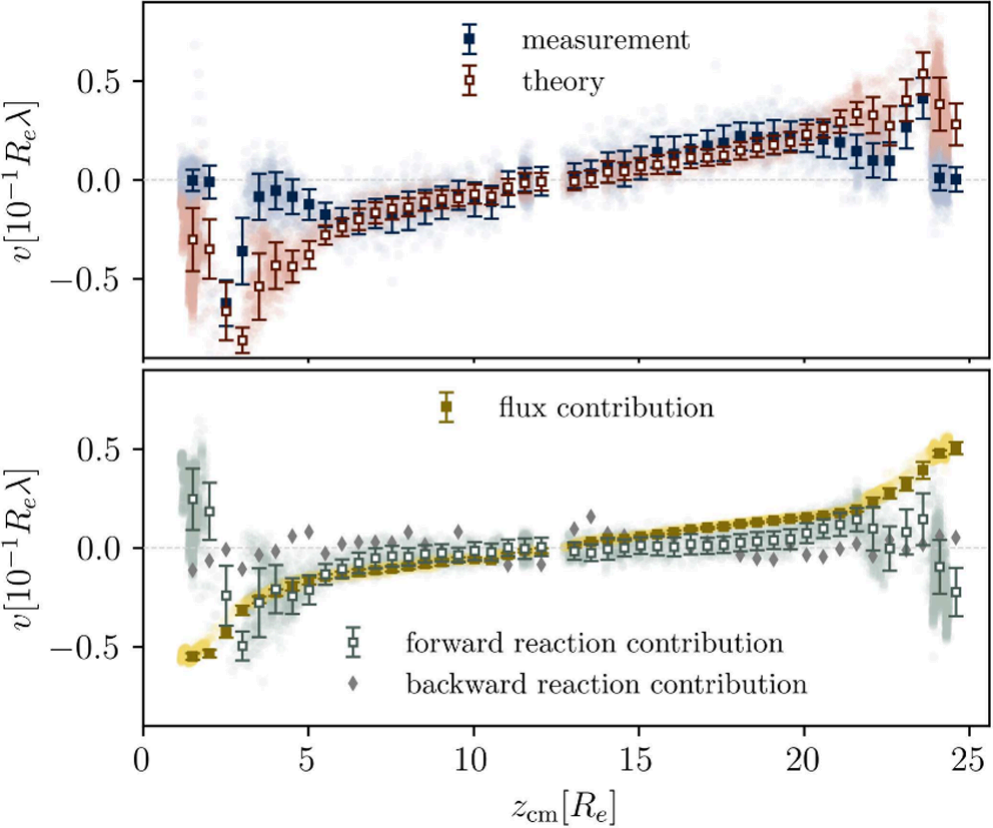
Velocities in *z*-direction, *v*, and their contributions plotted against the droplet centers, *z*_cm_. (Top) Mean measurement of droplet velocities using the HKCA (blue) and mean prediction of eq 4 (red). The individual results are plotted as dots in light colors underneath and the error bars indicate the spread among these. (Bottom) Decomposition of the prediction into the three contributions, flux contribution (yellow), forward reaction contribution (turquoise), as well as the backward reaction contribution (gray). For the former two we show individual droplet predictions in light colored dots, but omit this for the last one for clarity of the figure.

An analytical prediction of the concentrations is not available but we can use the measured concentration fields and concomitant fluxes, droplet radii, and Voronoi cells to compare the prediction of eq 4 with the simulation data for the droplet velocity, *v*. Figure 5 shows the measured droplet velocities, obtained by averaging over velocities of all droplets at position, *z* = *z*_cm_, and the theoretical prediction. The prediction matches the measurement in the bulk but deviates close to the source, where the prediction overestimates the velocity. From Figure 3 (a) we infer that the deviation occurs where droplets collide with the layer of shells and fuse – phenomena that are not considered in eq 4.

The lower panel of Figure 5 breaks down the prediction into its three contributions. The nonreactive flux contribution is the dominant driving force for movement toward the source. Inside the bulk, 5 ≤ *z*/*R*_e_ ≤ 20.6, the forward reaction contribution is slightly smaller and acts in the same direction. Close to the source, however, it becomes small and can act even in opposite direction, due to highly asymmetric accumulation volumes, Ω_*i*_, as described in detail in Sec. Reaction Contribution to Velocities of the SI. The backward reaction contribution is largely negligible.

#### Implicit Fuel

**Figure 6:**
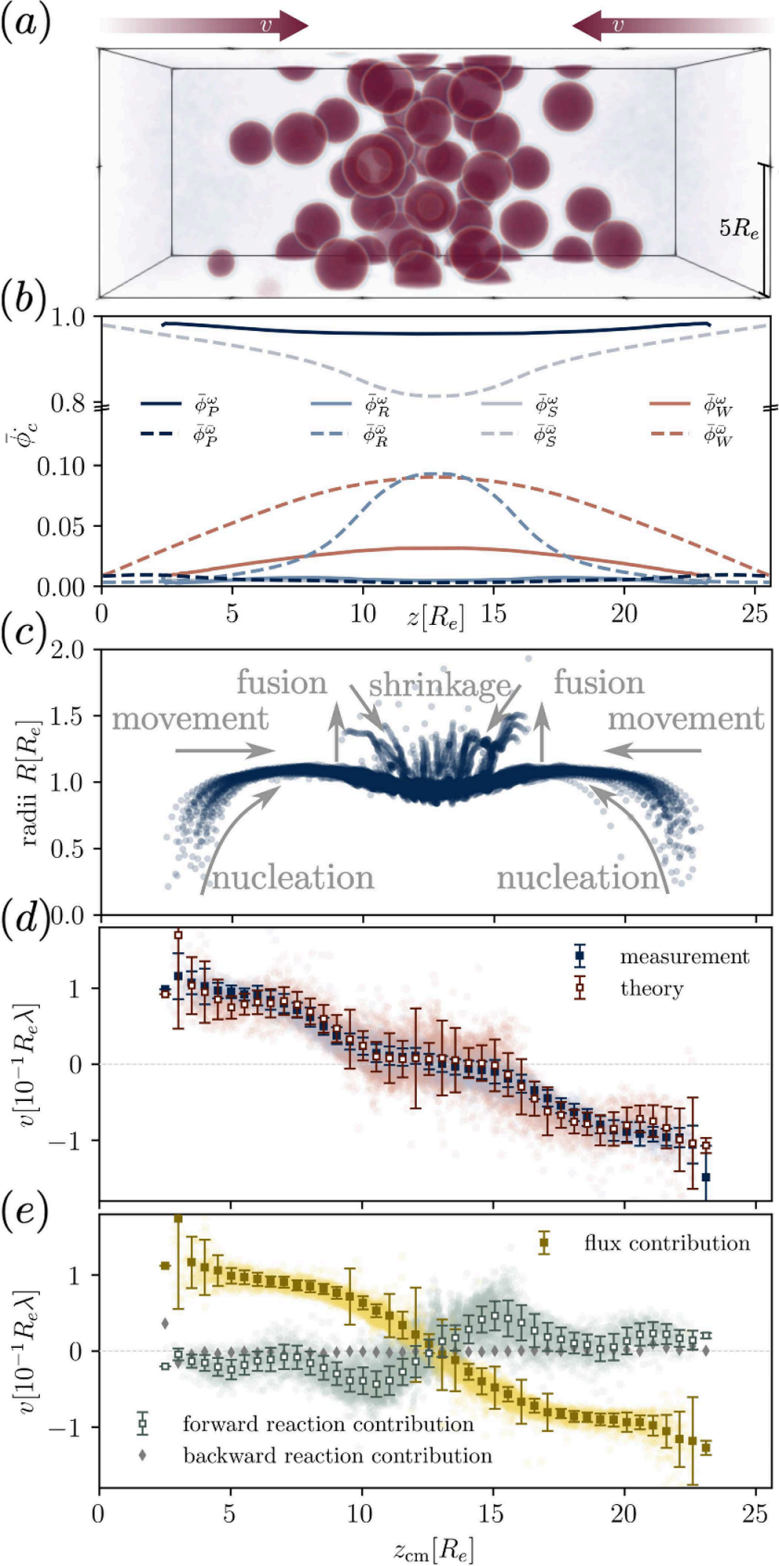
Simulation results for implicit treatment of fuel for reaction rate constant *r*_*b*_ = 0.08λ and neutral interaction χ_*PW*_ = 0. (a) Example morphology at *tλ* = 1000. The movement of condensates is indicated. (b) Laterally averaged concentration profiles inside,  (solid), and outside the droplets,  (dashed). (c) Droplet radii, *R*, as obtained from the HKCA. The dynamics seen in the simulations are indicated. (d) Measurements and predictions of vertical droplet velocity, *v*, plotted against the center of mass, *z*_cm_. (e) Three contributions of the prediction from eq 4 plotted against center of mass, *z*_cm_.

In the previous section waste was treated as part of the solvent, *i. e.*, strongly repulsive toward the product. Importantly, since the fuel source also acts as a waste (solvent) sink in this setup, the waste that is produced in the bulk diffuses toward the sink. This implies a waste-flux effect that is opposite to the one of fuel flux if there are more favorable interactions of waste toward the product. To show this, we explicitly treat waste with χ_*WP*_*N*_*P*_ = 0 but fuel implicitly, as part of the solvent. The forward reaction is then described by *R* + *S* → *P* + *W* and commences with a rate constant, that is ten times higher than before, *r*_*f*_ = 4*r*_*b*_, in order to keep the conversion rate comparable to the previous setup. The simulation results are shown in Figure 6 and the dynamics can be appreciated in the Supplementary Movie II. In contrast to the previous setup, there is a directed movement of droplets to the center of the domain. As droplets move toward the center collectively, large voids arise, in which the fuel commences to react with precursor. Product becomes supersaturated in this part of the solution and, eventually, a spherical droplet nucleates, which starts moving. In this scenario, droplets grow rapidly after nucleation and only become a little smaller before the finite size of the simulation cell gives rise to collision with oncoming droplets from the other side, Figure 6 (a). This dynamics can also be identified in the individual droplet radii as a function of their center of mass, presented in Figure 6 (c). At the center, fusion events result in droplets that become too large to be maintained externally by RDA and vacuolize, see Figure 6 (a). In the laterally averaged concentration profiles, shown in Figure 6 (b), one can see that, following the product droplets’ movement into the center, the precursor is enriched in the solution at the center. Waste also has the highest concentration in the solution at the center, whereas it is slightly lower inside the droplets, for entropic reasons. The inhomogeneous waste profile implies a flux of waste toward the sink on the boundaries, *z* = 0 and *L*. Finally, the local velocities are measured and compared to the theoretical prediction in Figure 6 (d), where the prediction matches the measurements. Figure 6 (e) dissects the prediction into its contributions: The nonreactive flux contribution stems from the waste flux from the center to the sink and its coupling to the product flux via incompressibility. This contribution sets the overall nature of the droplet movement. The forward reaction contribution is large close to the center and is opposed to the nonreactive flux contribution. It gives rise to the ’shoulder’ that is visible in the velocity profile at the center. The shape of the contribution is dictated by the strong asymmetry of accumulation volumes, where droplets have a significantly larger volume behind than in front, when colliding with the crowd of droplets at the center. Whereas the former contribution is largely independent of the droplet arrangement within the simulation cell, this contribution depends heavily on the arrangement and overall movement of droplets. Hence, the nature of the forward reaction contribution is determined by the nonreactive flux contribution. Again, the backward reaction contribution fluctuates around zero and is negligible.

### RDA-Formed Droplets with Explicit Fuel and Waste

The previous two scenarios showcased two diametrically opposed effects, movement of RDA droplets toward the fuel source, mainly due to a diffusive flux of fuel from the source into the bulk, and movement of these away from the waste sink, due to a diffusive flux of waste from the bulk to the sink. Treating now both components explicitly, with neutral or favorable interactions toward the product, and locating fuel source and waste sink in the same place will result in both effects working against each other. Indeed, if the two components have the same properties, *i. e.*, interactions and mobilities, both flux contributions cancel exactly. We detail this argument in Sec. Fuel and Waste Fluxes Cancel in the Symmetric Case of the SI and show supporting simulations. After an initial transient, when the fuel flux dominates because waste is not yet present, the movement of droplets toward the sides slows down and, eventually, completely halts. This can be observed in the Supplementary Movie III.

**Figure 7:**
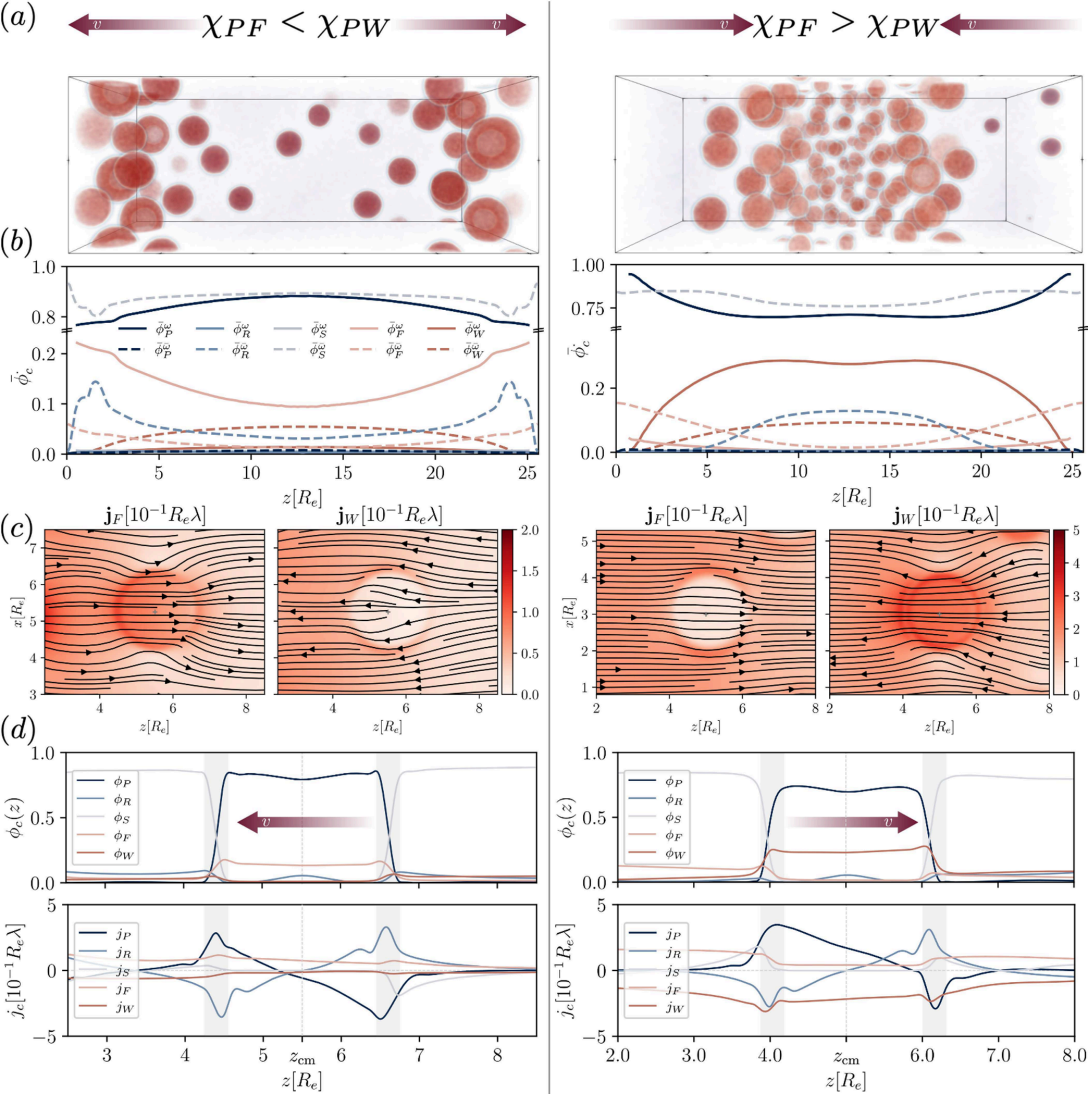
(a) Example morphologies for two interaction scenarios for reaction rate constant, *r*_*b*_ = 0.08λ. On the left, fuel has a higher affinity toward product than waste (χ_*PF*_*N*_*P*_ = −40, χ_*PW*_*N*_*P*_ = 0), leading to movement of product droplets toward the fuel source. On the right, waste has the higher affinity (χ_*PF*_*N*_*P*_ = 0, χ_*PW*_*N*_*P*_ = −40), leading to movement toward the center. (b) Temporally and laterally averaged concentration profiles, inside the droplets,  (solid), and outside,  (dashed). (c) (Noise-smoothed) Flux field of fuel, **j**_*F*_(**r**), and waste, **j**_*W*_(**r**), showing a droplet on the left side of the domain. (d) Concentration profiles (top) and flux profiles (bottom) through the center of the droplet of panel (c) along the *z*-direction. The direction of transport is indicated. The droplet interface is indicated in gray.

In natural systems, this symmetry between fuel and waste with respect to their interactions and mobility can be broken, allowing for an exquisite control over the directed motion of droplets. Therefore, let us consider different scenarios where either the fuel or waste has an affinity for the product, χ_*PF*_*N*_*P*_ < 0 or χ_*PW*_*N*_*P*_ < 0, whereas the other component interacts neutrally. The attractive component is enriched inside the product droplets which results in its flux contribution to dominate and, once again, we observe directed movement of the RDA droplets. Specifically, if χ_*PF*_ < χ_*PW*_, droplets move toward fuel source and waste sink. On the contrary, if χ_*PF*_ > χ_*PW*_ droplets move away from the waste source and fuel sink, toward the center of the domain. This is demonstrated in Figure 7 (a). Example morphologies are given for these two scenarios and the movement can be appreciated in the Supplementary Movies IV and V .

Figure 7 (b) shows temporally and laterally averaged concentration profiles, outside, , and inside, , of the droplets for the two different scenarios. Visibly, the component which has an affinity toward the product is significantly enriched inside the droplets, whereas the nonpreferential component is depleted. Consequently, the attractive component preferentially flows through the droplets, determining their directed motion. Figure 7 (c) demonstrates this behavior for the example of a single droplet for the two scenarios. Finally, Figure 7 (d) shows the concentration and flux profiles for a cut through the center of the example droplets of panel (c). The direction of movement is indicated by the arrows. Again, one can observe the enrichment or depletion of fuel or waste, as well as the slight enrichment of precursor in the droplet center. From the flux profiles, we can identify once more the three contributions: Driven by the forward reaction, there is an influx of product on the outside, potentially asymmetric. Driven by the backward reaction, precursor flows from the inside toward the outside, approximately spherically symmetric. Fuel and waste both flow through the droplet and their combined flux determines the backward flux of product in the droplet center. Visibly, there is  in both scenarios, leading to the corresponding droplet motion.

**Figure 8:**
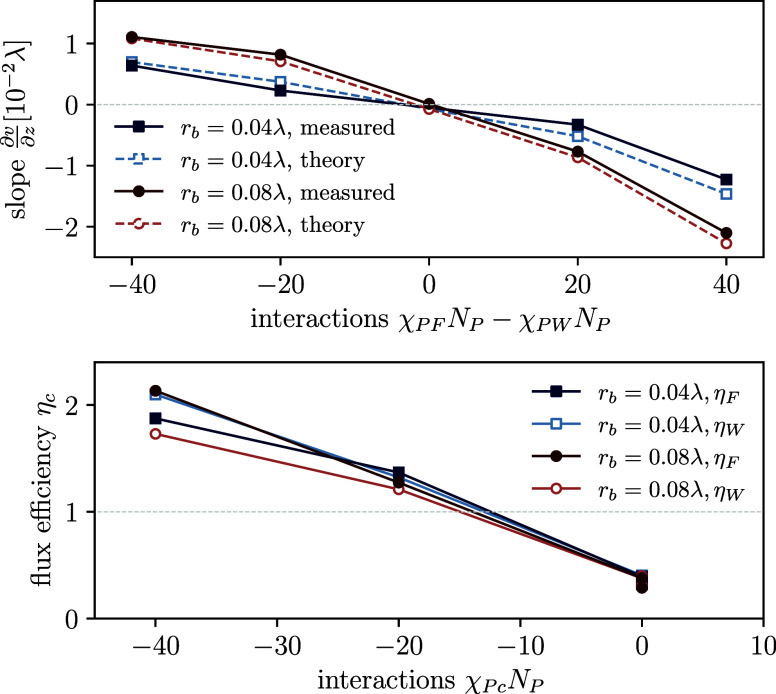
Top: Indicator for the droplet dynamics, the slope, *∂v*/*∂z*, of a linear fit through the velocity profiles in the bulk plotted against the interaction difference (χ_*PF*_ – χ_*PW*_)*N*_*P*_, where one of the interactions always vanishes and the other one is negative. Thus, for negative values fuel has an attraction toward the product and for positive ones waste is attractive. Bottom: Efficiency measurement, η_*c*_, of the components *c* = *F*, *W* for all systems.

The simulation results for a variation of interactions and reaction rate are depicted in full detail in Sec. Simulation Results for Explicit Fuel and Waste, where we show the radii, velocity measurement and theoretical comparison, as well as the individual contributions to the velocity for each parameter. We introduce a crude estimator for the droplet dynamics, by taking the slope of a linear fit through the velocity measurements in the bulk of the domain, 5 ≤ *z* ≤ 20.6*R*_e_. The linear fit is depicted in Figure S9 (b) of the SI. Its slope adopts positive values for movement toward the fuel source, while it becomes negative for droplet movement away from the source into the bulk. We compare the slope for the measurement and the theoretical estimation of eq 4 in Figure 8 (top). Clearly, the more attractive one of the components becomes, the faster the droplet movement becomes toward its source. A higher reaction rate also leads to faster movement, since concentration gradients become stronger. Moreover there is an asymmetry in the plot, indicating that we observe higher velocities in the case of waste-attractive scenarios than in the fuel-attractive ones.

Additionally we quantify the flux efficiency through the droplet by comparing the fuel and waste flux through the droplet to the laterally averaged flux (corresponding to the uniform flux of the system without droplets). This motivates us to define the efficiency, , for *c* = *F*, *W*. Figure 8 (bottom) shows the efficiency plotted against the respective interaction parameter. Clearly the attractive component, which is enriched inside the droplets experiences a significant efficiency increase. For the strongest attractions that we have probed, the flux increases by a factor of η_*c*_ ≈ 2 on the inside compared to the outside, while it is decreased to η_*c*_ ≈ 0.3 for nonpreferential interactions of fuel or waste.

To corroborate the continuum model results, we perform particle-based simulations of a soft, coarse-grained model for polymer melts, making use of the single-chain in mean field approximation.^59,60^ In Sec. Particle-based Simulations^61^ of the SI we show that we observe the same droplet dynamics and interaction dependencies.

### Multiple Droplet Types

**Figure 9:**
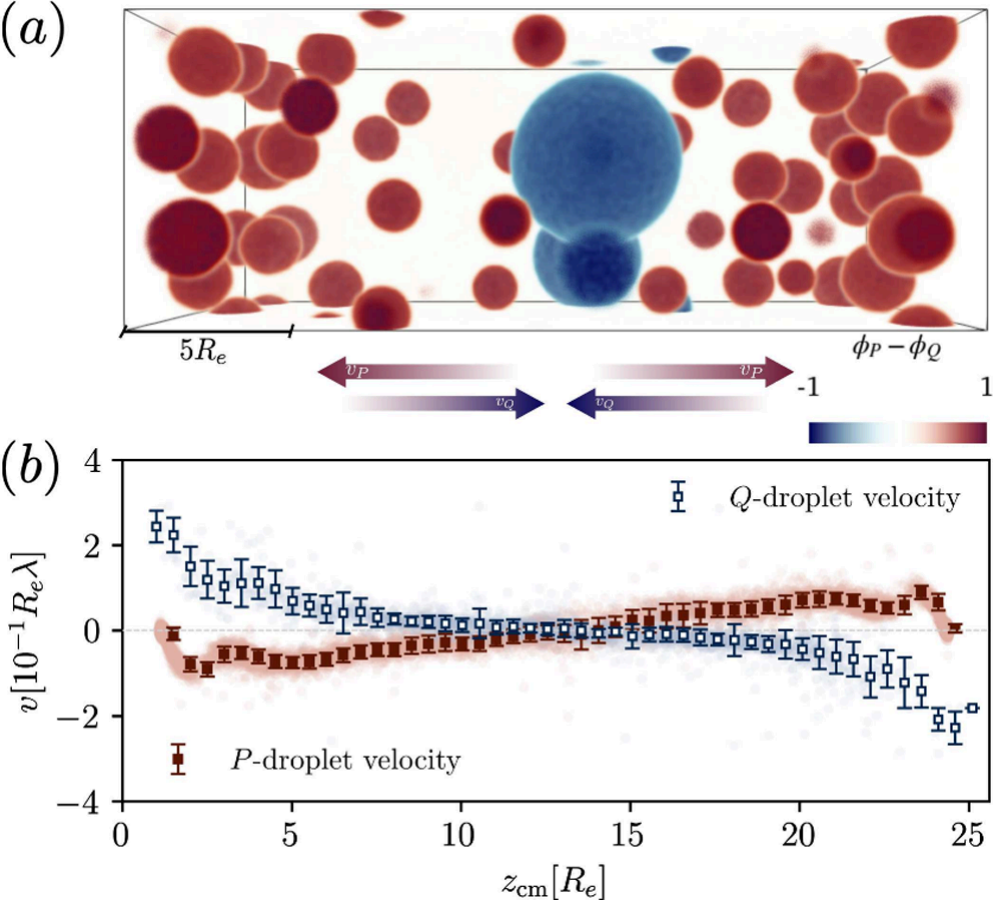
Simulations with additional waste-attractive component *Q*, which is not involved in the reaction cycle. (a) Example snapshot after the *Q*-rich droplets have moved to the center *tλ* = 500. (b) Velocity measurements of *P*- (red) and *Q*-rich (blue) droplets plotted against droplet position.

We note that in biological systems, there typically exists a plethora of phase separating quantities,^2,44,62,63^ inducing the formation of many moieties with entirely different compositions. Here we demonstrate that the existence of a reaction cycle can lead to movement of all of such droplets, whether these are involved in the reaction or not. All of this only depends on the interactions with fuel and waste. This can lead to a subset of droplets being driven toward the center, *i. e.*, the moieties that interact favorably with the waste, whereas another subset is driven toward the fuel source and waste sink, *i. e.*, the ones that interact favorably with the fuel. We show the phenomenon for two types of droplets with different compositions and interactions with fuel and waste. To this end, we introduce another macromolecular species, *Q*, that phase separates from solution but is not involved in the reaction cycle. Introducing an incompatibility between the two hydrophobic liquids, *P* and *Q*, results in the formation of two distinct droplet types. The parameters and simulation protocol is described in Sec. Multiple Droplet Types of the SI. Interacting favorably with the waste, the *Q*-rich droplets move toward the center of the domain and coalesce, while the *P*-rich droplets that are formed by RDA continue to nucleate in the center and move toward the source at the boundary of the simulation cell. The final *Q*-rich droplet is thus driven into a nonequilibrium state in which the diffusion away from the center is hindered such that it stays positioned in the center. Figure 9 (a) shows an example snapshot after all of the (passive) *Q*-rich droplets have been transported to the center, while the *P*-rich droplets stay dynamic without reaching a stationary state. The dynamics can be observed in Supplementary Movie VI. Figure 9 (b) shows the corresponding velocity measurements for the two droplet types, where the opposed movement becomes clear. Hence, this is a reliable but very primitive method for (proto-) cells, to organize their inside components.

### Growing Complex Reaction-Driven Assemblies

In the previous scenarios, we considered a simple phase-separating product, whose droplets did not exhibit internal structure and no free-energy barriers existed that could hinder merging of aggregates. Here, we will consider a reaction cycle, in which the product is amphiphilic, instead of simply hydrophobic. Such a system has been introduced in ref (52), illustrating the general, universal aspects of reaction-driven amphiphilic self-assembly. We model the amphiphilic product as a diblock copolymer, with a hydrophilic *A*-head and a hydrophobic *B*-tail. To do so, we employ the free-energy derived by Uneyama and Doi,^64−66^ which reduces to the previous free-energy for single-block molecules and to the Ohta-Kawasaki model^67^ close to the onset of microphase separation. The continuum model allows to blend different molecular architectures, which is required here.

We provide a description of the simulations and parameter details in Sec. Simulations of Amphiphilic Product Molecules of the SI and the dynamics can be appreciated in the Supplementary Movie VII. The amphiphilic product aggregates into micelles or vesicles. Most importantly the hydrophobic tails of the product are repulsive toward precursor, solvent, fuel, and waste, *i. e.*, there is no nonreactive flow across the hydrophobic aggregate core. However, the hydrophilic head groups, which form their own fluid domain surrounding the hydrophobic domain, interact favorably with the fuel and solvent, and there is a finite flux of these through this headgroup domain. In the absence of a concentration gradient, the stable morphology consists of stacked planar bilayers (lamellar phase) or vesicles. Forming such aggregates from an initially homogeneous solution, however, requires topological changes from micelles to vesicles or merging of aggregates that are hampered by a free-energy barrier. This distinguishes the behavior of self-assembled, amphiphilic aggregates from droplets that readily merge. Thus, in the absence of motion, amphiphilic systems are likely to become trapped in intermediate morphologies such as micelles (see Figure S11 of the SI).

If, however, fuel is locally refilled, the concomitant nonreactive fuel flux from the sides of the simulation cell toward the center will generate an opposite flux of aggregates with fuel-attractive headgroup toward the boundaries of the simulation cell. This motion allows for a slow growth of the aggregates as they move toward higher fuel concentration.

**Figure 10:**
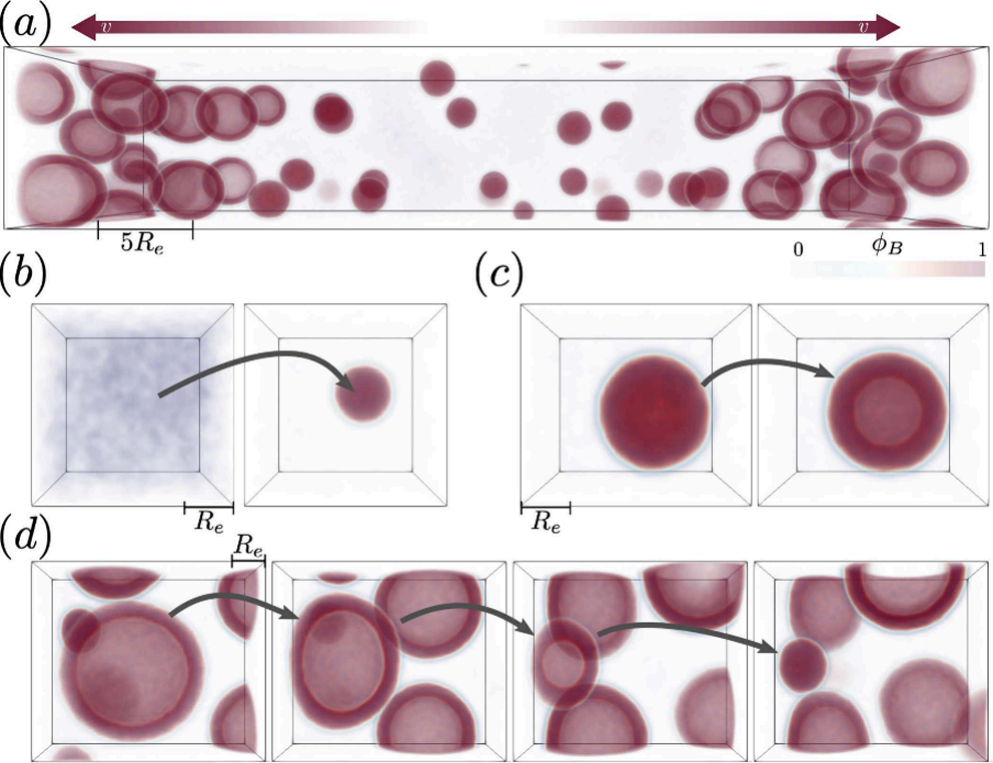
Example snapshot of a system with amphiphilic product, depicting the hydrophobic product component, *B*. Micelles nucleate at the center of the simulation cell, move toward the sides (as indicated by the arrows) and form vesicles, once large enough. At the sides, high competition for product leads to shrinkage of the vesicles. The individual topological changes are depicted below: (b) nucleation of micelle after supersaturation of the solution with product, (c) micelle to vesicle transformation by flip-flopping of amphiphiles to the inside, and (d) shrinking of vesicles near the fuel source.

Micelles nucleate at the center of the simulation cell. As they move toward the fuel source, they gradually grow and a hydrophilic center can form inside the micelle core, facilitated by precursor that has recently reverted. This hydrophilic center then acts as a nucleation site for amphiphiles to flip-flop to the inside and form a vesicle,^52,68^ similar to the mechanism of vacuolization of RDA-formed droplets in Sec. Implicit Waste. The nucleation process to a micelle is depicted in Figure 10 (b) and the transformation from micelle to vesicle in Figure 10 (c). Hence, the directed movement inside the fuel-concentration gradient allows to circumvent free-energy barriers and gradually build more complex structures, which would not form otherwise.

At the fuel source (*i. e.* simulation-cell boundary), the vesicles collide but do not fuse. Since the closely packed vesicles are deprived of their accumulation volume they slowly disintegrate, as seen in Figure 10 (d).

## Conclusion

In this work, we explored the diffusiophoresis of liquid condensates both, passive and driven by chemical reaction cycles. We showed that the directed movement of droplets within externally maintained concentration gradients differs from diffusiophoresis of colloids, because there exists a finite flux within the droplet. This flux through the droplets can be depleted or enhanced, depending on the interactions of the droplet material, and it dictates the direction of the droplet’s movement. By virtue of the incompressibility constraint, the movement is opposed to the flux of fuel or waste through the droplet and is independent of the aggregate size.

Concentration gradients naturally occur in systems that are driven by chemical reactions due to the local sources of fuel and sinks of waste. For reaction-driven assembly (RDA)-formed droplets, the flux-mediated movement persists. Analytically, we also found additional contributions to the droplet velocity that arise from the asymmetry of the reactions in the droplet’s surrounding. These turned out to be corrections, for the cases studied.

Importantly, the fluxes of fuel and waste generated by the chemical reactions will direct the movements of aggregates, through which fuel or waste flow, independently of whether or not they participate in the reaction cycle.

A concentration gradient and the concomitant movement of droplets or aggregates provides a temporal change of environment that modulates the nucleation and growth of aggregates. This facilitates transformations of the aggregate structure, *e. g.* vacuolization of droplets or the transformation from micelles to vesicles that are otherwise hindered by large free-energy barriers.

In a broader biological context, this mechanism of directed movement offers a straightforward yet robust and versatile means of transporting organelles, with or without membranes, within finite domains – akin to (early) cellular systems. For cells we have estimated velocities up to (10–100)nm/s. The effect may also guide the design of synthetic reaction-driven assemblies. The direction of transport for these organelles is distinct for each type and sensitively depends on their interactions with fuel and waste constituents.

## Methods

Within the continuum model, normalized local concentrations, ϕ_*c*_(**r**) of solvent, reactant (precursor), product, fuel and waste, *c* = *S*, *R*, *P*, *F*, *W*, are taken as order parameters, that determine the free-energy. We employ the Flory–Huggins-de Gennes free-energy functional of eq 1. In the free energy, the first term is the Flory–Huggins entropy of mixing,^69,70^ the second one is a square-gradient penalty to create a finite interface width. The concentration-dependence of the coefficient of the square-gradient theory is motivated by the Lifshitz entropy, characterizing the narrow interfaces of polymers. Components interact via binary interactions whose strength is determined by the Flory–Huggins parameters χ_*cc*′_ in the third term. The system is incompressible, which is enforced by the last term with the Lagrange multiplier, π(**r**).

Given the free-energy functional, we obtain the chemical potentials as the functional derivatives with respect to the concentrations, , which yields
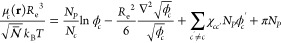
5

Gradients in the chemical potentials give rise to fluxes that minimize the free energy,
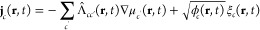
6where the Onsager matrix is chosen diagonal, , since incompressibility is enforced by the free-energy functional. In the following, we define . λ_*P*_*R*_*e*_^2^ is the diffusion coefficient of the product molecule, *P*. Its self-diffusion time, λ_*P*_^-1^, is taken as the reference time scale. The factor λ_*c*_ determines the mobility of component, *c*, which we take the same for all components, λ_*c*_ = λ_*P*_≕λ. Furthermore, ξ_*c*_(**r**, *t*) is Gaussian thermal noise. Its moments are determined by the fluctuation–dissipation relation^71,72^
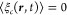
7
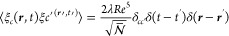
8Without the reactions, concentrations are locally conserved, hence the equilibration follows model-B dynamics.^50^ Meanwhile, insertion of fuel to the system enables reactions that drive the system out of equilibrium.^37,73^ This yields the time evolution of the concentrations
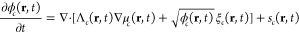
9The reaction rates are *s*_*c*_ = *s*_*f*,*c*_ + *s*_*b*,*c*_ + *s*_source,*c*_ + *s*_sink,*c*_ from forward and backward reaction, source and sink. The first two are given by
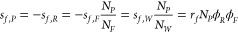
10
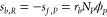
11*i.e.*, the forward reaction creates product, diminishes reactant and fuel, and accumulates waste, leading to transient assembly provided that the initial conditions contain fuel. To maintain a nonequilibrium steady state, however, fuel needs to be inserted into the system locally. Here, we consider a cuboidal domain with periodic boundary conditions, where a fuel source and waste sink are located inside slabs at *z* = 0 and *z* = *L*_*z*_. Inside these slabs solvent is replaced by fuel or waste by solvent, respectively,
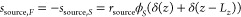
12
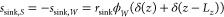
13

Thereby the material conversion of fuel to waste in the bulk is compensated, allowing for the emergence of a statistically stationary nonequilibrium state.

The product, *P*, phase separates from solution, forming droplets, whereas the precursor, *R*, is a solute to the solvent. Hence, there is a repulsion of product to solvent and precursor. The backward reaction rate constants are chosen to create droplet radii of only a few *R*_e_. The forward reaction rate constant is scaled, such that the forward and backward reaction probability for the reactant (precursor) are identical at a small fuel concentration, ϕ_*F*_ = 0.025. This justifies the choice of parameters in Table 1.

**Table 1:** Choice of Parameters in the Flory–Huggins–de Gennes Model^a^

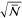	*N*_*R*/*P*_	*N*_*S*/*F*/*W*_	χ_*PS*_*N*_*P*_	χ_*PR*_*N*_*P*_	χ_*P F*/*W*_*N*_*P*_	*r*_*b*_	*r*_*f*_	*r*_source_	*r*_sink_
2500	10	1	50	20	0, −20, −40	(4, 8) · 10^–2^λ	40*r*_*b*_	*R*_b_*R_e_*/4	10*λR*_e_

a For parameters that are varied, multiple values are given.

The size of the simulation cell is chosen *V* = 9.6*R*_e_ × 9.6*R*_e_ × 25.6*R*_e_, with a spatial resolution of 10 grid cells per *R*_e_. We introduce a wall for the droplets, which is modeled via an external field. The external field is introduced by adding the term *∑*_*c*_*f*_ext,*c*_(*z*)*N*_*P*_ϕ_*c*_(**r**, *t*) to the integrand of eq 1. By using a large positive value for precursor and product close to the origin, *z* = 0 and *L*_*z*_, we suppress their densities,
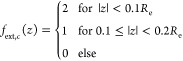
14for *c* = *R*, *P* and *f*_ext,*c*_ = 0 for *c* = *S*, *F*, *W*. This hinders clogging of source and sink by aggregates and collisions of incoming aggregates, with no qualitative effect on the overall dynamics of the system.

All simulations are started from homogeneous initial conditions with mean concentrations of , ϕ_*S*_ = 0.855, and . We start with very small but nonzero mean concentrations for the latter three species to avoid numerical instabilities in the continuum model simulations. Notice that the overall concentration of reactant plus product stays constant, . If not stated otherwise, we run the simulations up to *tλ* = 1000 with a time step of Δ*tλ* = 4 · 10^–5^, *i. e.*, 2.5 · 10^7^ time steps. Measurements and averages are conducted over the last *tλ* = 500 when the systems have reached a statistically stationary state.

The coupled, partial differential equations are numerically solved using the pseudospectral method with semi-implicit time-stepping implemented on a graphics processing unit (GPU), as described in more detail in ref (52). Additional information about the software usage, availability and analysis steps is compiled in Sec. Analysis Procedure of the SI.

## Supporting Information Available

Approximate analytical calculation for the droplet velocity, additional numerical results, the numerical implementation, the analysis procedure, as well as descriptions of the supplementary movies (PDF)
Movies I–VII (ZIP)
