# Supplementary material for: Reaction-Driven Diffusiophoresis of Liquid Condensates: Potential Mechanisms for Intracellular Organization
Source: ACS Nano. 2024 Jun 14;18(26):16530–44. doi: 10.1021/acsnano.3c12842 (PMC11223496; doi:10.1021/acsnano.3c12842)
Supplement: Supplementary file 1 — nn3c12842_si_001.pdf [file nn3c12842_si_001.pdf]

Supporting Information to Reaction-driven Diffusiophoresis of Liquid Condensates: Potential Mechanisms for Intra-cellular Organization

Gregor Häfner[†,‡] and Marcus Müller[*,†,‡]

†Georg-August Universität Göttingen, Institut für Theoretische Physik, Friedrich-Hund Platz 1, 37077 Göttingen, Germany

‡Max Planck School Matter to Life, Jahnstraße 29, 69120 Heidelberg, Germany

E-mail: mmueller@theorie.physik.uni-goettingen.de

Theoretical Prediction of Velocities

In the following, we analytically calculate a prediction for the droplet velocity that takes averaged concentration profiles, as well as locally averaged radii and accumulation volumes from the simulations as input. We start with a one-dimensional (1D) calculation and subsequently extend the result to the three-dimensional (3D) case.

First, we evaluate the fluxes, satisfying the condition of incompressibility. The flux of component c is given by Equation 6 which, ignoring thermal noise, we rewrite to

$$\mathbf{j}_c(\mathbf{r}) = -\Lambda_c(\mathbf{r})\nabla\left(\mu_c^0(\mathbf{r}) + \pi(\mathbf{r})N_P\frac{\sqrt{\bar{\mathcal{N}}}k_{\mathrm{B}}T}{R_e^3}\right) \tag{S1}$$

where the bare chemical potentials μ_c^0 are the chemical potentials without the contribution of

the Lagrange multiplier.[52,61] Incompressibility demands the total concentration be constant and hence the total flux vanishes, $\sum_c \mathbf{j}_c = 0$. This allows us to determine the Lagrange multiplier exactly,

$$\nabla\pi(\mathbf{r}) N_P \frac{\sqrt{\bar{\mathcal{N}}} k_{\mathrm{B}} T}{R_e^3} = -\frac{\sum_{c'} \Lambda_{c'} \nabla \mu^0_{c'}}{\sum_{c'} \Lambda_{c'}}. \tag{S2}$$

Plugging this back into Equation S1, we obtain

$$\mathbf{j}_c = \sum_{c'} \frac{\Lambda_c \Lambda_{c'}}{\sum_{\tilde{c}} \Lambda_{\tilde{c}}} \nabla \left(\mu^0_c - \mu^0_{c'}\right) = \frac{1}{\sum_{\tilde{c}} \lambda_{\tilde{c}} \phi_{\tilde{c}}} \sum_{c'} \left(\lambda_{c'} \phi_{c'} \mathbf{j}^0_c - \lambda_c \phi_c \mathbf{j}^0_{c'}\right), \tag{S3}$$

with the bare fluxes

$$\mathbf{j}^0_c = -\Lambda_c \nabla \mu^0_c = -\lambda_c R_{\mathrm{e}}^2 \left(\frac{N_P}{N_c} \nabla \phi_c - \frac{R_{\mathrm{e}}^2}{6} \phi_c \nabla \left(\frac{\nabla^2 \sqrt{\phi_c}}{\sqrt{\phi_c}} \right) + \sum_{c' \neq c} \chi_{cc'} N_P \phi_c \nabla \phi_{c'} \right). \tag{S4}$$

With this form of coupling the bare fluxes, the incompressibility constraint is fulfilled.

Now we continue with the evaluation of droplet velocities, starting with a 1D droplet in concentration gradients. In this section, we consider the dynamics in the vicinity of an individual droplet and omit the index i running over these. We assume that the droplet with radius R positioned at $z_{\mathrm{cm}} = 0$ at time $t = 0$ accumulates product material from its closest surroundings (*i. e.*, 1D analog of Voronoi cell), $\Omega = [z_{\Omega^-}, z_{\Omega^+}]$. Since the droplet moves with velocity, v, we exploit that the concentration profiles stay roughly constant in the (instantaneously) co-moving frame, up to small changes upon moving through the concentration gradient, and denote the stationary quantities by a prime, *e. g.*, $\phi_c(z,t) = \phi'_c(z')$ for $z' := z - vt$. We assume interfaces to be infinitely sharp. Then, the integral of $\phi_P(z,t)$ over the droplet domain, $\omega = [-R+vt, R+vt]$ yields the total droplet material, which we assume to be instantaneously conserved in the co-moving frame. This corresponds to the assumption that the droplet instantaneously adopts the size that balances loss of product material on the inside and total production in the accumulation volume. Integrating over a

domain larger than the droplet domain, in order to include the interfaces and discontinuities in the fluxes, then taking the limit we obtain

$$\lim_{\epsilon\to 0}\int_{-R-\epsilon}^{R+\epsilon} \mathrm{d}z'\,\phi_P'(z') = \lim_{\epsilon\to 0}\left[[\phi_P'(z')'z]_{-R-\epsilon}^{R+\epsilon} - \int_{-R-\epsilon}^{R+\epsilon}\mathrm{d}z'\,\partial_{z'}\phi_P'(z')z'\right] \tag{S5}$$

$$= \lim_{\epsilon\to 0}\left[R[\phi_P'(R+\epsilon)+\phi_P'(-R-\epsilon)] + \frac{1}{v}\int_{-R-\epsilon}^{R+\epsilon}\mathrm{d}z'\,\partial_t\phi_P'(z')z'\right] \tag{S6}$$

At this point, we make the approximation (ICA) that product material, which is created inside the accumulation volume, instantaneously condenses onto the droplet's interface. Moreover, the droplet shape remains spherical independent of the decay of droplet material on the inside and the condensation on the outside.

In 1D, this modifies the time-evolution, Equation 2, for each droplet region, including its accumulation volume, to

$$\begin{aligned}\partial_t\phi_P(z,t) = -\partial_z j_P^{\mathrm{nr}}(z,t) + \delta(z+R-vt)\int_{z_{\Omega-}}^{vt}\mathrm{d}z\,[s_P(z,t)]\zeta(z-z_{\mathrm{cm}})\\ + \delta(z-R-vt)\int_{vt}^{z_{\Omega+}}\mathrm{d}z\,[s_P(z,t)]\zeta(z-z_{\mathrm{cm}})\end{aligned} \tag{S7}$$

where $\delta(\cdot)$ denotes Dirac's delta function and $\zeta(z-z_{\mathrm{cm}})$ is an assignment function, which accounts for the finite lifetime of product molecules, *i. e.*, the ratio of the rate at which product molecules reach the interface and the production rate in the accumulation volume. It is estimated for the 3D-case below. Since the driven reactions are now replaced by surface sources and sinks, we retain only the 'non-reactive' flux, *i. e.*, the precursor-flux contribution to the product flux, $-j_R$, is replaced by surface sources,

$$j_P^{\mathrm{nr}}(\mathbf{r}) \approx j_P + j_R \approx \begin{cases} 0 & \text{for } \mathbf{r}\notin\omega \\ -(j_F+j_W) & \text{for } \mathbf{r}\in\omega \end{cases}, \tag{S8}$$

using that outside the droplet, $\mathbf{r}\notin\omega$, the product concentration almost vanishes $\phi_P\approx 0$.

With this, the above calculation becomes

$$
\begin{aligned}
\lim_{\epsilon\to 0}\int_{-R-\epsilon}^{R+\epsilon}\mathrm{d}z'\,\phi_P'(z') = \lim_{\epsilon\to 0}\Bigg[& R[\phi_P'(R+\epsilon)+\phi_P'(-R-\epsilon)] - \frac{1}{v}\int_{-R-\epsilon}^{R+\epsilon}\mathrm{d}z'\,z'\partial_z j_P^{\mathrm{nr}\prime}(z') \\
& + \frac{1}{v}\int_{-R-\epsilon}^{R+\epsilon}\mathrm{d}z'\,z'\delta(z'+R+\epsilon)\int_{z'_{\Omega-}}^{0}\mathrm{d}z'\,\zeta(z-z_{\mathrm{cm}})s_P'(z') \\
& + \frac{1}{v}\int_{-R-\epsilon}^{R+\epsilon}\mathrm{d}z'\,z'\delta(z'-R-\epsilon)\int_{0}^{z'_{\Omega+}}\mathrm{d}z'\,\zeta(z-z_{\mathrm{cm}})s_P'(z')\Bigg] \qquad \text{(S9)} \\
= \lim_{\epsilon\to 0}\Bigg[& \frac{R}{v}\underbrace{[v\phi_P'(R+\epsilon)-j_P^{\mathrm{nr}\prime}(R+\epsilon)+v\phi_P'(-R-\epsilon)-j_P^{\mathrm{nr}\prime}(-R-\epsilon)]}_{=0\,(\mathrm{ICA})} \\
& - \frac{1}{v}\int_{-R-\epsilon}^{R+\epsilon}\mathrm{d}z'\,[j_F'(z')+j_W'(z')] \\
& - \frac{R}{v}\int_{z'_{\Omega-}}^{0}\mathrm{d}z'\,\zeta(z-z_{\mathrm{cm}})s_P'(z') + \frac{R}{v}\int_{0}^{z'_{\Omega+}}\mathrm{d}z'\,\zeta(z-z_{\mathrm{cm}})[s_P'(z')]\Bigg] \qquad \text{(S10)}
\end{aligned}
$$

where the second term was integrated by parts. Taking the limit, then results in an expression that quantifies the velocity in terms of integrals over the product concentration and the fluxes.

$$
\begin{aligned}
v\int_{z_{\mathrm{cm}}-R}^{z_{\mathrm{cm}}+R}\mathrm{d}z\,\phi_P(z) = & -\int_{z_{\mathrm{cm}}-R}^{z_{\mathrm{cm}}+R}\mathrm{d}z\,[j_F(z)+j_W(z)] \\
& + \frac{1}{2}\left(\int_{z_{\mathrm{cm}}}^{z_{\Omega+}}\mathrm{d}z\,\zeta(z-z_{\mathrm{cm}})s_P(z) - \int_{z_{\Omega-}}^{z_{\mathrm{cm}}}\mathrm{d}z\,\zeta(z-z_{\mathrm{cm}})s_P(z)\right). \qquad \text{(S11)}
\end{aligned}
$$

There are two contributions: The first arises from the fluxes inside the droplet and the second stems from the reaction/condensation in the droplet's accumulation volume. The second contribution is illustrated in Figure S1.

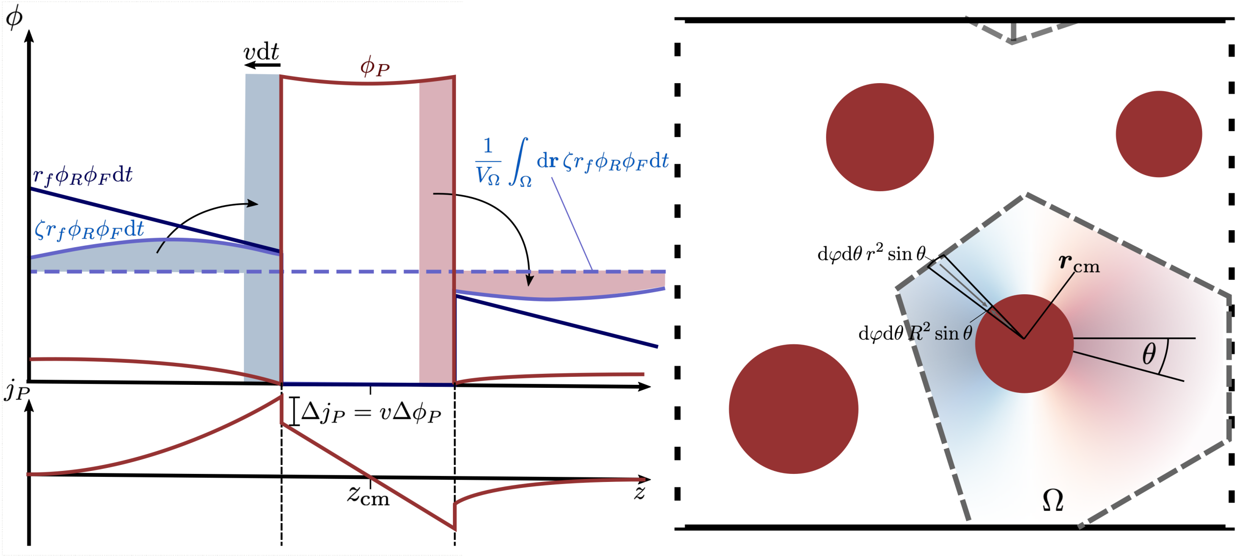

Figure S1: Sketch of the contribution of production effects in fuel and precursor concentration gradients. Left: Visualization of the second contribution of Equation S11. What is produced as excess to the average in Ω (for infinitesimal time step $\mathrm{d}t$) leads to movement of the interface, $v\mathrm{d}t$ on the left to the left. Correspondingly, the lack of production compared to the average leads to a retraction of the interface on the right (top). In the corresponding (reactive) product flux profiles there are jumps at the interfaces corresponding to the movement, which arises from an excess production on the left (bottom). The reactive flux is set to 0 in the instantaneous condensation approximation and replaced by surface source and sink shown at the top. Right: In 3D, droplets (red) are assigned an accumulation volume, Ω, shown by dashed lines, which corresponds to the Voronoi domain. The contribution of the local production, $\zeta(|\mathbf{r}-\mathbf{r}_{\mathrm{cm}}|)\cos(\theta)$, to the velocity in z-direction, $v_z = v$ is indicated by the heat map (blue negative, red positive).

Let us generalize the above calculation to 3D. We assume a spherical droplet of radius R moving at velocity $\mathbf{v} = v\hat{\mathbf{e}}_z$, where $\hat{\mathbf{e}}_z$ is the unit vector, pointing in z-direction. The droplet domain is described by $\omega = \{\mathbf{r}|\ ||\mathbf{r}-\mathbf{v}t|| < R\}$, and its surface denoted by $\partial\omega$. Again, we approximate all product that is produced within the accumulation volume to condense instantaneously onto the droplet's surface. This modifies the evolution equation to

$$\partial_t\phi'_P(\mathbf{r}') = -\nabla\cdot\mathbf{j}_P^{\mathrm{nr}\prime}(\mathbf{r}',t) + \delta(r'-R)\int_0^{r'_\Omega(\theta',\varphi')}\mathrm{d}r'\,\frac{r'^2}{R^2}\zeta(r')s'_P(r',\theta',\varphi',t) \tag{S12}$$

where the co-moving position is $\mathbf{r}' = \mathbf{r} - \mathbf{v}t$, and r', θ', φ' denote the spherical coordinates with respect to the droplet's center. We assume the droplet morphology (shape and size) be

stationary in the co-moving frame. Here and in the following, all functions of the argument $\mathbf{r}'$ are denoted by a prime. The fraction that appears in the integral is the result of the fraction of area elements from accumulation in the domain, $r'^2 \sin\theta' \mathrm{d}\theta' \mathrm{d}\varphi'$, and deposition at the sphere's surface, $R^2 \sin\theta' \mathrm{d}\theta' \mathrm{d}\varphi'$. Hence, first, we assume that molecules reach the droplet at the angle, at which they have been produced in the accumulation volume. More accurately, the molecules will perform a random motion, before reaching the droplets surface. We will phenomenologically reintroduce this effect below and account for it in the assignment function $\zeta(|\mathbf{r}-\mathbf{r}_{\mathrm{cm}}|)$. Hence in 3D, we have

$$\int_{\omega'} \mathrm{d}\mathbf{r}'\, \phi'_P(\mathbf{r}') = \underbrace{\int_{\partial\omega'} \mathrm{d}\Gamma' \phi'_P(\mathbf{r}') z'(\hat{\mathbf{e}}_{z'} \cdot \hat{\mathbf{e}}_{r'})}_{=:\mathrm{b.t.}} - \int_{\omega'} \mathrm{d}\mathbf{r}' z' \underbrace{\hat{\mathbf{e}}_{z'} \cdot \nabla' \phi'_P(\mathbf{r}')}_{=\partial_{z'}\phi'_P(\mathbf{r}-\mathbf{v}t)} \tag{S13}$$

$$= \mathrm{b.t.} + \frac{1}{v}\int_{\omega'} \mathrm{d}\mathbf{r}' z' \partial_t \phi'_P(\mathbf{r}') \tag{S14}$$

$$\overset{\text{(S12)}}{=} \mathrm{b.t.} + \frac{1}{v}\int_{\omega'} \mathrm{d}\mathbf{r}' z' \left[-\nabla' \cdot \mathbf{j}_P^{\mathrm{cm}\prime}(\mathbf{r}') + \delta(r'-R) \int_0^{r'_\Omega(\theta',\varphi')} \mathrm{d}r'\, \frac{r'^2}{R^2} \zeta(r') s'_P \right] \tag{S15}$$

$$= \mathrm{b.t.} - \frac{1}{v}\int_{\omega'} \mathrm{d}\mathbf{r}' z' \nabla' \cdot \mathbf{j}_P^{\mathrm{nr}\prime}(\mathbf{r}') + \frac{1}{v}\int_0^{2\pi} \mathrm{d}\varphi' \int_0^{\pi} \mathrm{d}\theta' \int_0^{R} \mathrm{d}r' r'^3 \sin\theta' \cos\theta' \times \delta(r'-R) \int_0^{r'_\Omega(\theta',\varphi')} \mathrm{d}r'\, \frac{r'^2}{R^2} \zeta(r') s'_P \tag{S16}$$

$$= \frac{1}{v}\int_{\partial\omega'} \mathrm{d}\Gamma'\, z' [\mathbf{v}\phi'_P(\mathbf{r}') - \mathbf{j}_P^{\mathrm{nr}\prime}(\mathbf{r}')] \cdot \hat{\mathbf{e}}_{r'} + \frac{1}{v}\int_{\omega'} \mathrm{d}\mathbf{r}' \hat{\mathbf{e}}_{z'} \cdot \mathbf{j}'_P(\mathbf{r}') + \frac{R}{v}\int_0^{2\pi} \mathrm{d}\varphi' \int_0^{\pi} \mathrm{d}\theta' \int_0^{r'_\Omega(\theta',\varphi')} \mathrm{d}r'\, r'^2 \sin\theta' s'_P \zeta(r') \cos\theta' \tag{S17}$$

$$= -\frac{1}{v}\int_{\omega'} \mathrm{d}\mathbf{r}' \hat{\mathbf{e}}_{z'} \cdot [\mathbf{j}'_F(\mathbf{r}') + \mathbf{j}'_W(\mathbf{r}')] + \frac{R}{v}\int_{\Omega'} \mathrm{d}\mathbf{r}'\, s'_P \zeta(r') \cos\theta'. \tag{S18}$$

where the boundary term vanishes, having formally taken a spherical integration domain with radius $R \to R + \epsilon$ with a small $\epsilon > 0$ and the subsequent limit $\epsilon \to 0$, as was explicitly

shown in the 1D case. We obtain the final estimate for the velocity,

$$v \int_{\omega} \mathrm{d}\mathbf{r}\, \phi_P(\mathbf{r}) = -\int_{\omega} \mathrm{d}\mathbf{r}\, [j_F(\mathbf{r}) + j_W(\mathbf{r})] + R \int_{\Omega} \mathrm{d}\mathbf{r}\, [r_f N_P \phi_R \phi_F - r_b N_P \phi_P] \zeta(|\mathbf{r} - \mathbf{r}_{\mathrm{cm}}|) \cos\theta \,, \tag{S19}$$

where $j_c := \mathbf{j}_c \cdot \hat{\mathbf{e}}_z$ and again, θ is the angle between $\mathbf{r} - \mathbf{r}_{\mathrm{cm}}$ and the z-axis, as illustrated in Figure S1. The figure also presents a visualization of the second contribution to the velocity.

Additionally, we account for the random, diffusive motion of molecules before reaching the droplet via the assignment $\zeta(|\mathbf{r} - \mathbf{r}_{\mathrm{cm}}|)$. Without it, molecules would be assumed to reach the droplet at the angle, at which they react to become product. In reality the random motion will blur the position of entry into the droplet. To quantify this factor, we performed simple simulations of a Wiener process, starting at position $\mathbf{r}_0 = r\hat{\mathbf{e}}_z$, *i. e.*, $\theta_0 = 0$ and measured the entry angle $\theta_{\mathrm{arrival}}$ at which the molecules would reach a sphere of radius R, located at the origin. This allows to calculate the average angle contribution $\langle \cos\theta_{\mathrm{arrival}} \rangle$ and their probability to reach the droplet, $\mathcal{P}_{\mathrm{arrival}}$. The assignment function is then given by $\zeta(r) = \mathcal{P}_{\mathrm{arrival}}(r) \langle \cos\theta_{\mathrm{arrival}} \rangle(r)$. The result is shown in Figure S2. Starting within close

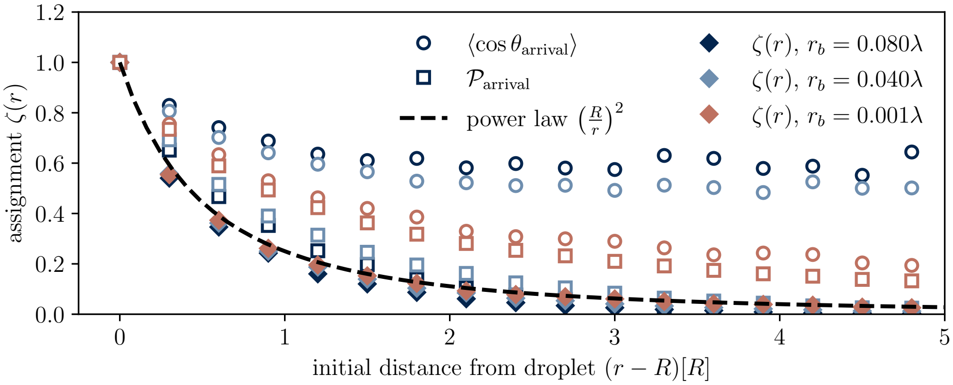

Figure S2: Assignment function, $\zeta(r)$ plotted against distance from the droplet interface, $r - R$, for different reaction rates, which changes the lifetime of product molecules. Additionally, we show the two contributions, arrival probability, $\mathcal{P}_{\mathrm{arrival}}$ (squares), and directional contribution, $\langle \cos\theta_{\mathrm{arrival}} \rangle$ (spheres). For the different life times the assignment function collapses onto the power law $\zeta(r) = \frac{R^2}{r^2}$ (dashed).

vicinity of the droplet, molecules have a high probability to reach the surface, $\mathcal{P}_{\mathrm{arrival}} \approx 1$,

and do so at an entry angle close to the starting one, $\theta_{\text{arrival}} \approx \theta_0$, *i. e.*, $\cos\theta_{\text{arrival}} \approx 1$. For starting positions further away from the droplet, the arrival probability is lower and the spread of the entry angle is larger, which also diminishes the average $\langle \cos\theta_{\text{arrival}} \rangle$. Comparing different reaction rates (corresponding to life times), while the arrival probability is lower for smaller life times, the entry angle, θ_{arrival}, is spread less such that the assignment functions for all reaction rates (across a wide range of values) collapse onto a universal curve which is nicely described by an effective power law, $\sim r^{-2}$. This motivates the assignment function

$$\zeta(r) = \begin{cases} 1 & \text{for } r < R \\ \left(\frac{R}{r}\right)^2 & \text{else.} \end{cases} \tag{S20}$$

Notice that while the assignment function does not explicitly depend on the reaction rate anymore, it still does implicitly because droplet sizes, R, are smaller at higher reaction rates.

1D Passive Droplets in External Concentration Gradients

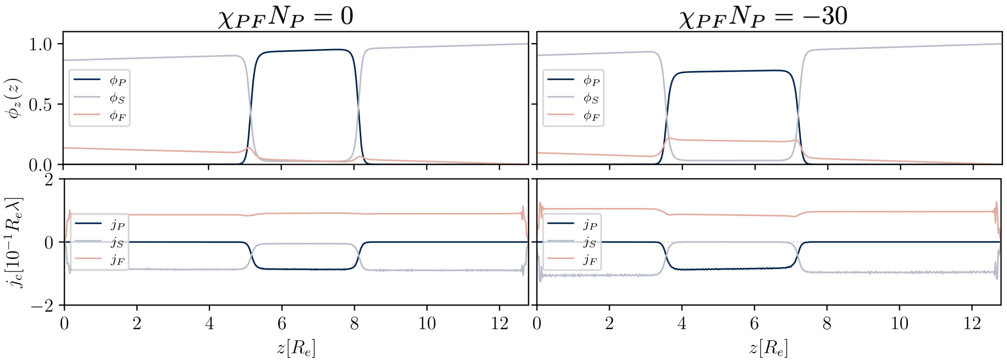

Figure S3: Simulation result of the 1D passive droplets in external concentration gradients, for two parameter combinations and without thermal noise. In both cases there is a strong repulsion $\chi_{PS}N_P = 30$, with neutral or attractive interactions between F-solvent and droplet material, as indicated at the top. We show a snapshot at $t\lambda = 60$ after placing the droplet at the center of the simulation cell, $z_{\text{cm},t=0} = 12.4R_{\text{e}} = \frac{Lz}{2} - 0.4R_{\text{e}}$. Given are concentration profiles (top) and fluxes (bottom).

In this section, we compare the 3D passive droplets in external concentration gradients of Sec. Passive Droplets in External Concentration Gradients of the main text to their 1D

counterpart. As we have seen, in 3D the flux can flow around the droplet in the case where the droplet is unfavorable for the F-solvent, or preferentially through the droplet, in the case where the droplet region is attractive for it. This is at the expense of the regions surrounding the droplet laterally, where the F-solvent flux is in- or decreased, respectively. In 1D such a geometric modification of the flux fields is not possible. In such a scenario the flux has to be constant in the bulk regions and jumps at the interface, if it moves with velocity v and there is a jump in concentration, for all components. Indeed this is what we observe in the 1D simulations. This is shown in Figure S3. Again, the F-solvent is depleted inside the droplet for neutral interactions, because of the asymmetry in molecular volume, while it enriched inside the droplet for favorable interactions. This is the same is in the 3D case. However, the F-flux behaves opposite to the 3D case: It is higher on the inside than on the outside for the depleted concentration and it is reversed for the enriched concentration. This relation is simply dictated by the movement of the droplet, which demands $\Delta j_F = v\Delta\phi_F$. This explains the higher F-flux inside the droplet for the case $\chi_{PF}N_P = 0$ than on the outside (at the left interface: $\Delta\phi_F \approx -0.05$, $v \approx -0.1R_\text{e}\lambda$, $\Delta j_F \approx 0.005R_\text{e}\lambda$), and correspondingly the lower one inside for $\chi_{PF}N_P = -30$ ($\Delta\phi_F \approx 0.14$, $v \approx -0.12R_\text{e}\lambda$, $\Delta j_F \approx -0.018R_\text{e}\lambda$). Compared to the 3D case, here, all material of F and S necessarily must flow through the droplet. Thus, the droplet is a bottleneck in this scenario and influences the (otherwise constant)[1] flux in the whole simulation cell. That way the droplet is still a little bit faster in the attractive scenario than in the neutral one.

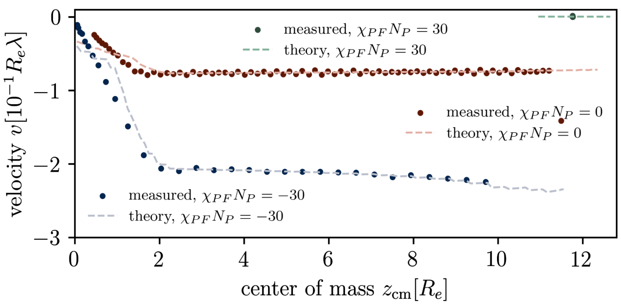

Figure S4: Droplet velocity in z-direction, v, plotted against center of mass, $z_{\rm cm}$, both as measured from simulation and with the theoretical prediction using the measured fluxes and concentrations inside the droplet.

Velocity of Passive Droplets in External Concentration Gradients

For the simulations of Sec. Passive Droplets in External Concentration Gradients we can measure the droplet velocity and compare it the theoretical prediction above, which relates it to the fluxes and concentrations.

To do so, we perform the Hoshen-Kopelman cluster analysis (HKCA),[53,54] where a droplet is defined by the threshold $\phi_P > 0.5$. This allows to measure the droplets center of mass, $\mathbf{r}_{\rm cm}$, and thereby its velocity using two consecutive time steps. Figure S4 depicts the measured velocities as a function of the center of mass. The velocities are approximately constant inside the bulk and approach zero once the droplet overlaps with the F-source, *i. e.*, once its center of mass reaches $z = R$, with R being the droplet's radius. We compare the measured velocities to the theoretical prediction, which is worked out for the general case of reaction-driven assembly (RDA)-formed droplets in fuel and waste concentration gradients in Sec. Theoretical Prediction of Velocities, resulting in Equation S19. For the chemically passive droplets, $r_f = r_b = 0$, and assuming the concentration profiles vary slowly across the droplet volume, this simply reduces to $v = \frac{j_P(\mathbf{r}_{\rm cm})}{\phi_P(\mathbf{r}_{\rm cm})}$. Making use of the cluster analysis, we

[1] Note that the movement of the droplet, with other F-concentrations than in the surroundings implies a change of mean concentration in the system that changes the concentration profiles surrounding it and hence the flux is not strictly constant but skewed. This is slightly visible in Figure S3 with opposite skewedness for the two cases.

average the fluxes and concentrations within the droplets at every time step and use these averages to predict v. The result is plotted in Figure S4 as dashed lines and matches the measurements.

Nucleation of RDA-formed Droplets

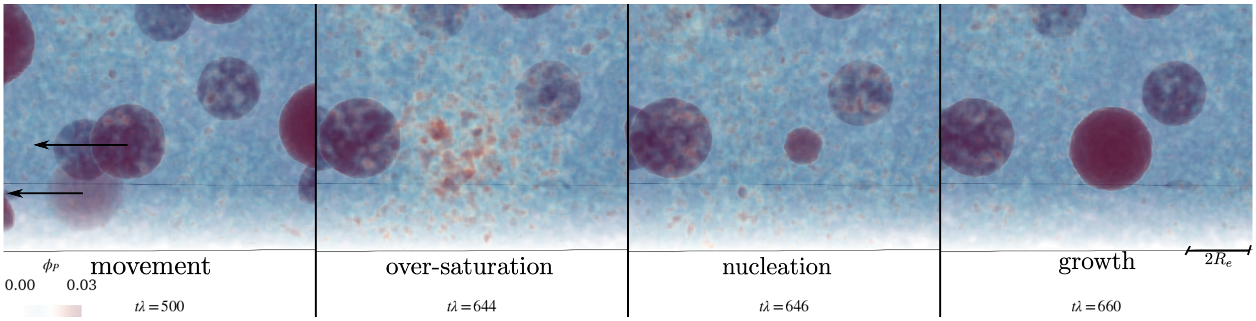

Figure S5: Nucleation dynamics of RDA-formed droplets for the setup with implicit waste of Sec. Implicit Waste in the main text. The different dynamics are indicated.

Here, we briefly demonstrate how RDA-formed droplets nucleate. We show this on the example system of Sec. Implicit Waste of the main text. The droplets move towards the sides of the simulation cell, towards higher fuel concentrations. In this setup, the collective movement creates large voids between droplets in which the forward reaction commences. This creates more product in the solution than can diffuse into the surrounding droplets or can revert into the precursor state. Thus, the product concentration in these void begins to rise until it comes close to the spinodal concentration. When this happens, a droplet can stochastically nucleate and such a nucleation event becomes the probable the higher the super-saturation $\phi_P(\mathbf{r}) - \phi_{P,\text{binodal}}$. Eventually, a new droplet nucleates, which quickly adsorbs the super-saturated product from its surrounding and grows to the stationary size. Such a nucleation event is shown in Figure S5.

Note that the nucleation happens close to the (mean-field) spinodal,[51] *i. e.*, when the free-energy barrier for the nucleation becomes very small (*i. e.* on the order of $k_\text{B}T$). The binodal was measured at $\phi_{P,\text{binodal}} = 0.002$ (with noise) and the mean-field spinodal at

$\phi_{P,\text{spinodal}} = 0.01$. This is where we typically observe the nucleation process, *i. e.* at high supersaturation. Hence, formation of droplets occurs in our simulations at the transition between (classical) nucleation and spinodal decomposition.

Choice of Boundary Conditions

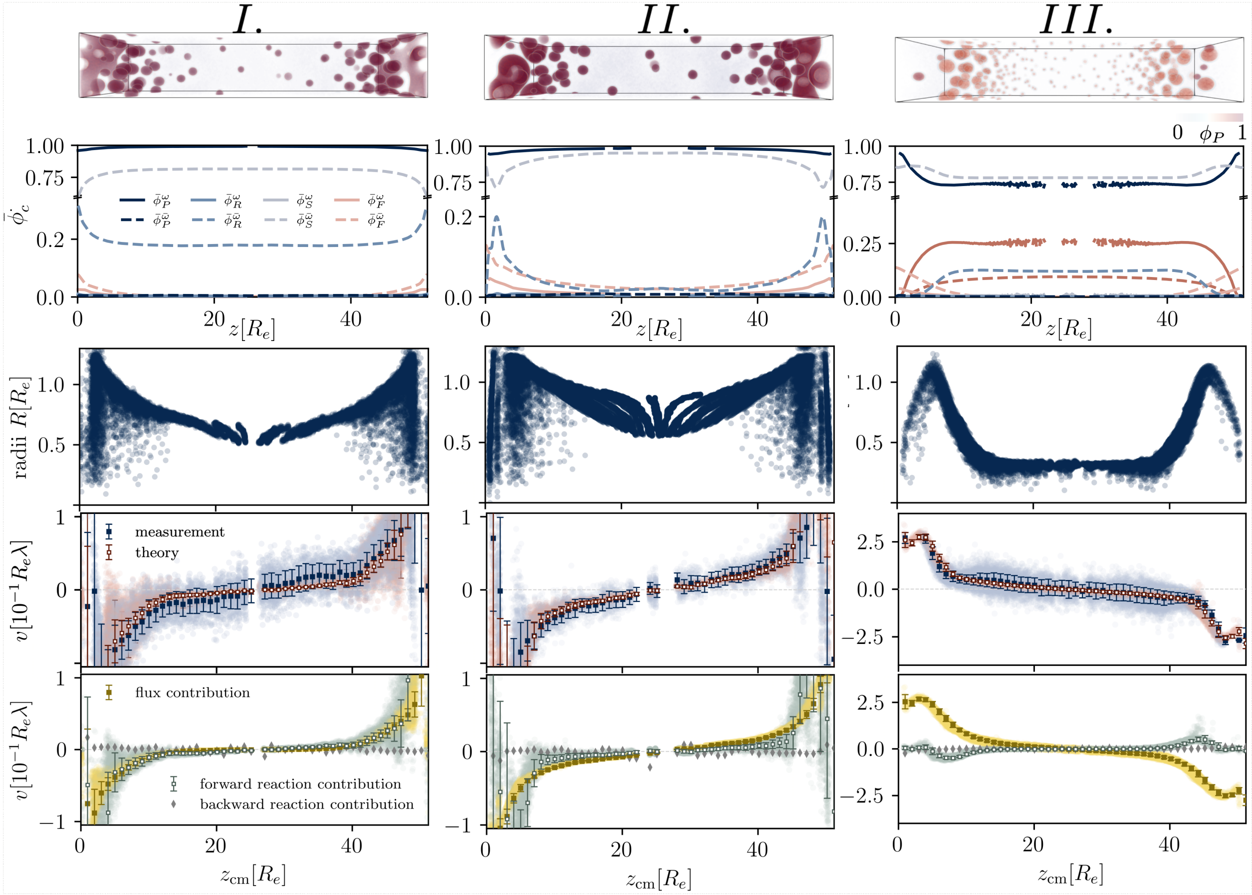

Figure S6: Simulation results for various systems, numbered as described in the text. For each column, the top panel shows an example snapshot of the product concentration field. In the second row, we show the temporally and laterally averaged concentration profiles inside (solid) and outside (dashed) of the droplets. The third row displays the measured droplet radii, plotted against their center of mass. The fourth row shows measured and theoretically predicted velocities plotted against center of mass. Individual measurements are shown as dots and the local averaged is marked with errorbars, indicating the standard deviation of the local distribution. Finally, the fifth panel does the same for the three contributions of the theoretical prediction.

In this section, we discuss different simulation-cell architectures and the resulting dy-

namics. We are considering three scenarios that differ from the simulations in the main text and compare these. The three scenarios are

I. Implicit treatment of waste with fully periodic boundary conditions, *i. e.*, no external field at the boundaries of the simulation cell. Additionally, we take a large z-dimension, $L_z = 51.2R_\mathrm{e}$, and double the initial precursor concentration, $\bar{\phi}_R + \bar{\phi}_P = 0.25$. The missing external field allows for droplets to cover the source of fuel, and thus a larger source rate constant, $r_\mathrm{source} = 0.1R_\mathrm{e}\lambda$, is necessary.

II. Implicit treatment of waste with larger simulation-cell size, $L_z = 51.2R_\mathrm{e}$, but external boundary field. The larger size of the simulation cell justifies a doubled source rate constant, $r_\mathrm{source} = 0.02R_\mathrm{e}\lambda$. All other parameters are kept the same as in the main text.

III. Explicit fuel and waste with an affinity of product towards waste, $\chi_{PW} = -40$, and a larger simulation-cell size of $L_z = 51.2R_\mathrm{e}$. All other parameters are unchanged compared to the main text.

The simulation results for the three system are shown in Figure S6. For system I, implicit fuel with a large simulation cell, more precursor and no wall, we observe the nucleation in the center of the system and subsequent directed movement towards the closest fuel source. Instead of stopping in front of the source, droplet freely move to the sides of the simulation cell, where they fuse with oncoming droplets. This allows fusion to large structures that regularly reach beyond the periodic boundary conditions. Allowing the movement above the source also allows to block it which periodically leads to depletion of fuel in the system, reducing the product concentration until the source is free, again. This undesirable dynamics justifies the external boundary field that was applied in all other simulations. Additionally, the large simulation cell allows nucleation of droplets far away from the source. Here the droplets do not grow as quickly as in the case of a smaller simulation cell. Droplets stay small while they are far away from the source and only gradually grow upon approaching

it. This becomes visible when looking at the droplet radii plotted against their center of mass. From the laterally averaged concentrations inside and outside of the droplets we can see that the precursor concentration is high in all of the solution. Measuring the velocity as a function of the center of mass results in a similar curve as the one from the main text, nicely matched by the theoretical prediction. Looking at the individual contribution to the prediction, the bottom panel, we realize that in this scenario, the reaction contributions are just as large as the flux contributions.

System II is closer to the system of the main text, with the difference of a doubled simulation-cell size and doubled refilling rate of the fuel, while we keep the polymer concentration low, $\bar{\phi}_R + \bar{\phi}_P = 0.125$. Due to the larger size of the simulation cell, we observe, again, a region of small droplet density in the center, where droplets that do nucleate stay small and gradually grow upon approaching the source. In comparison to system I, the precursor concentration becomes low in the center, while the fuel concentration does not fall off as fast. Compared to the smaller system size of the main text, the product is more concentrated at the walls, which results in larger structures that also regularly span beyond the periodic boundaries laterally. Again, the velocity prediction matches the measurements. In this scenario the reaction contribution is comparable to the flux contribution, however smaller in the bulk.

System III features fuel and waste explicitly, with an affinity of product towards waste. This scenario is tailored to show what happens if the simulation cell is larger than the length scale of the waste concentration profile and thus too large for droplets to approach in the center. As visible in the example snapshot and the radii plot of Figure S6, droplets nucleate close to the fuel source. From here they quickly move towards the center. At approximately $z \approx 10R_\mathrm{e}$ and $z \approx L_z - 10R_\mathrm{e}$ multiple droplets are closely packed, in full correspondence to the scenario with small simulation-cell size. When droplets approach this region, their accumulation volume becomes asymmetric, with small volume closer the center and large volume closer to the source. This leads to large forward reaction contributions opposing the

flux contribution, and the droplets are slowed down. In this region the droplets adopt the largest sizes, while they shrink as they move forward from here farther away from the source. After moving through the densely packed region, the droplets distance from each other while flux contribution drives them towards the center. The droplets continuously shrink until they reach their smallest possible size, dictated by the interface width ($R \approx 0.3R_{\mathrm{e}}$), and at this point dissolve. In this scenario, the velocity measurements also display the 'shoulder', the curve obtaining a small gradient in the center. Here it is not only a result of the jamming and the reaction contribution opposing the flux contribution but also the flux contribution itself displays this behavior because the waste concentration becomes saturated with a vanishing gradient at the center.

These three scenarios demonstrate the robustness of the mechanism, which qualitatively does not depend on the boundary conditions of the simulation cell. Quantitatively, however, the dynamics depends on those details offering opportunities to tailor the behavior.

Reaction Contribution to Velocities

We have shown in the main text that the forward reaction contribution strongly varies, depending on the system's morphology, *i. e.*, the arrangement of droplets and the distribution of precursor and fuel molecules. The movement that can be produced by the forward reaction is the result of an asymmetry in product that flows into the droplet. This can stem either from concentration gradients of fuel and precursor (*i. e.* their products) or from an asymmetry in the accumulation volume.

For the two examples of implicit waste or fuel of Sections Implicit Waste and Implicit Fuel of the main text, let us have a look at the statistics of the accumulation volumes and the averaged concentrations. For this we perform the Voronoi tessellation of the domains for each time step and locally average the distinct domains. Assuming cylindrical symmetry of the averaged domains, the result is presented in the insets of Figure S7. Clearly, the

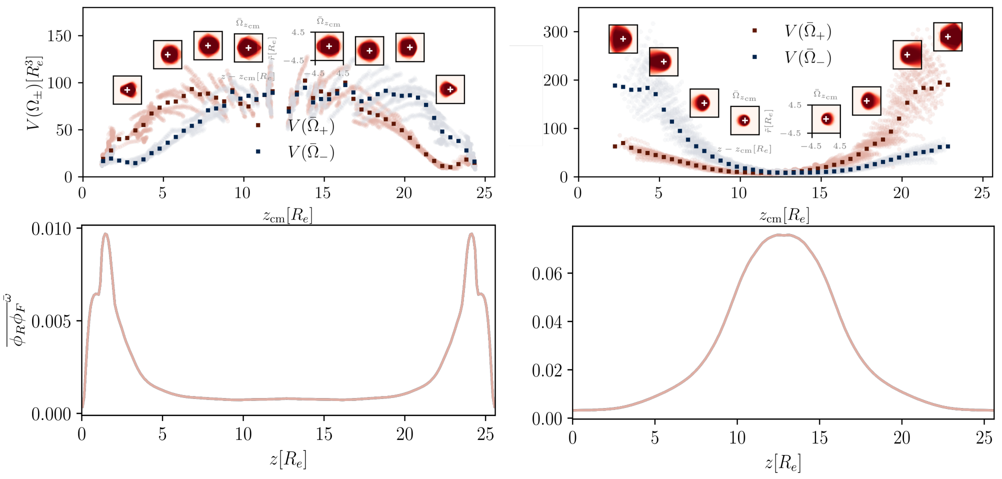

Figure S7: Measurement results for the system of implicit waste (left) and implicit fuel (right). In the top row, we show the mean volume of the accumulation domains in front of, $V(\Omega_-)$, and behind, $V(\Omega_+)$, the center of mass. The insets show the (locally) averaged domains, assuming cylindrical symmetry ($\tilde{r} = \sqrt{x^2 + y^2}$). The center of mass is marked as a cross to see the horizontal asymmetry in these. In the bottom row we show the temporally and laterally averaged product of concentrations $\phi_R\phi_F$, which are proportional to the product of the forward reaction (proportionality factor is, $r_f N_P = 3.2 N_P \lambda$ on the left and $r_f N_P = 0.32 N_P \lambda$ on the right).

accumulation volumes are asymmetric around the center of mass of the droplets: For implicit fuel and movement towards the sides of the simulation cell, the accumulation volumes are larger farther away from the source. In the opposite case, implicit fuel and movement towards the center, the accumulation volumes are larger at the side that is closer to the waste sink. This motivates analyzing the volume of the accumulation domains in front of the center of mass, $V(\Omega_-)$ for $z < z_{\rm cm}$, and behind it, $V(\Omega_+)$ for $z > z_{\rm cm}$. The result is depicted in the top row of Figure S7. The bottom row shows the temporally and laterally averaged product of fuel and precursor concentrations outside of the droplets, $\overline{\phi_R\phi_F}^{\bar{\omega}}$. In the case of implicit waste and movement towards the side, the precursor concentration is also higher at the sides of the simulation cell, as is the fuel concentration, and there is a high gradient of the product concentration. Hence, within a infinitesimal volume around droplet, the production is higher closer to the source than further away from it. It is the opposite for the scenario where droplets move towards the center, although the fuel concentration (solvent in this scenario) still is highest at the sides. Hence the two contributions to the reaction

contribution oppose each other. The result, the green lines of Figures 5 and 6, is that the reaction contribution works in the direction of the flux contribution in the first case, while it works in opposite direction of it (away from the center) in the case where droplets move away from the waste sink.

These two scenarios are representative also of the full systems, where both, fuel and waste, are treated explicitly.

Fuel and Waste Fluxes Cancel in the Symmetric Case

When interactions and mobilities of fuel and waste are the same, their effects on the droplets cancel exactly in the statistically stationary state, such that the droplets do not move.

For this argument, consider a system where no phase separation occurs. For simplicity we only consider fuel, waste and solvent, treating precursor and product implicitly, and analyze the resulting solvent flux. We take all mobilities to be the same, $\lambda_c = \lambda \, \forall c$ and all interactions to vanish, $\chi_{cc'} = 0 \, \forall c, c'$. Moreover, we disregard thermal fluctuations. For the fuel component, the time evolution becomes

$$\begin{aligned} \partial_t \phi_F &= \frac{\lambda R_e^5}{\sqrt{\bar{\mathcal{N}}} k_{\mathrm{B}} T} \nabla \cdot \left[\phi_F \phi_S \nabla (\mu_F - \mu_S) + \phi_F \phi_W \nabla (\mu_F - \mu_W) \right] - r_f(\mathbf{r}) \phi_F & \text{(S21)} \\ &= \lambda R_e^2 \nabla^2 \phi_F - r_f(\mathbf{r}) \phi_F & \text{(S22)} \end{aligned}$$

where $r_f(\mathbf{r}) \stackrel{\wedge}{=} r_f \phi_R(\mathbf{r})$ is now a general local forward reaction rate, and in the second line we consider only small concentration gradients, allowing us to disregard the square-gradient penalty in the chemical potential. The time evolution of the waste is analogous,

$$\partial_t \phi_W = \lambda R_e^2 \nabla^2 \phi_W + r_f(\mathbf{r}) \phi_F. \quad \text{(S23)}$$

In the stationary state, the resulting solvent flux takes the form

$$\mathbf{j}_S = -(\mathbf{j}_F + \mathbf{j}_W) = \lambda R_e^2 \nabla(\phi_F + \phi_W) \overset{\text{(S22),(S23)}}{=} 0. \tag{S24}$$

Since waste and fuel flux cancel exactly, the main contribution to the droplet velocity vanishes.

Indeed, such a system shows an arrest in movement and approaches a stationary state where large droplets arrange on a lattice structure close to the interface and droplet sizes decrease further away from the source. Here, the classical size-control mechanism of such RDA systems becomes visible, dictating the lattice spacing, and size of droplets then being determined by the (local) mean concentration of product, which is smaller further away from the fuel source. The simulation results and analysis are shown in Figure S8. While the flux inside the droplets vanishes entirely, the reaction contributions do not, *i. e.*, the asymmetry in accumulation volumes does not entirely counter the asymmetric production because of concentration gradients. Here, the deviation between prediction and measurement is again prominent close to the source.

Simulation Results for Explicit Fuel and Waste

For explicit treatment of fuel and waste we vary the interactions of fuel and waste with the product, χ_{PF} and χ_{PW}, and the reaction rate constants, r_b. Performing all measurements, as before, we obtain the simulation results in Figure S9. Panel (a) shows the measured droplet radii plotted against the droplets' center of mass. Qualitatively one can observe nucleation in the center for the attractive-fuel case. $\chi_{PF} < 0$, while the nucleation happens near the source in the opposite case, $\chi_{PW} < 0$. For large affinities one can also observe fusion events where the droplets move to, while such events are not observable for intermediate attractions (lower velocities). The droplets become larger for smaller a reaction rate constant, $r_b = 0.04\lambda$. The velocity measurements and their theoretical comparisons are plotted in

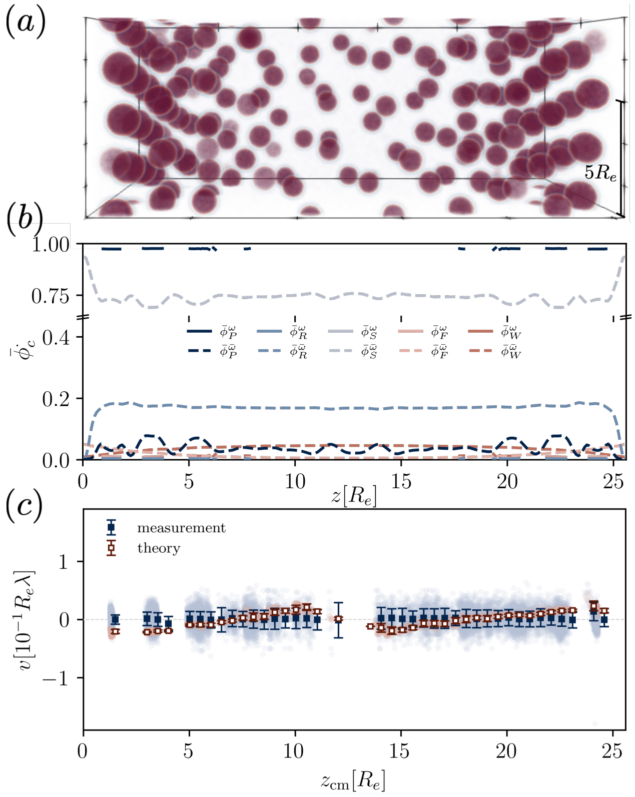

Figure S8: Simulation results for explicit treatment of fuel and waste with symmetric interactions, $\chi_{PF} = \chi_{PW} = 0$, showing (a) an example snapshot after time $t\lambda = 1900$, after the system has become stationary. (b) The averaged concentration profiles inside and outside the droplets. Due to the lattice structure, in between the droplet layers, there is also no data on the inside concentrations. (c) Measured and theoretical velocities plotted against center of mass.

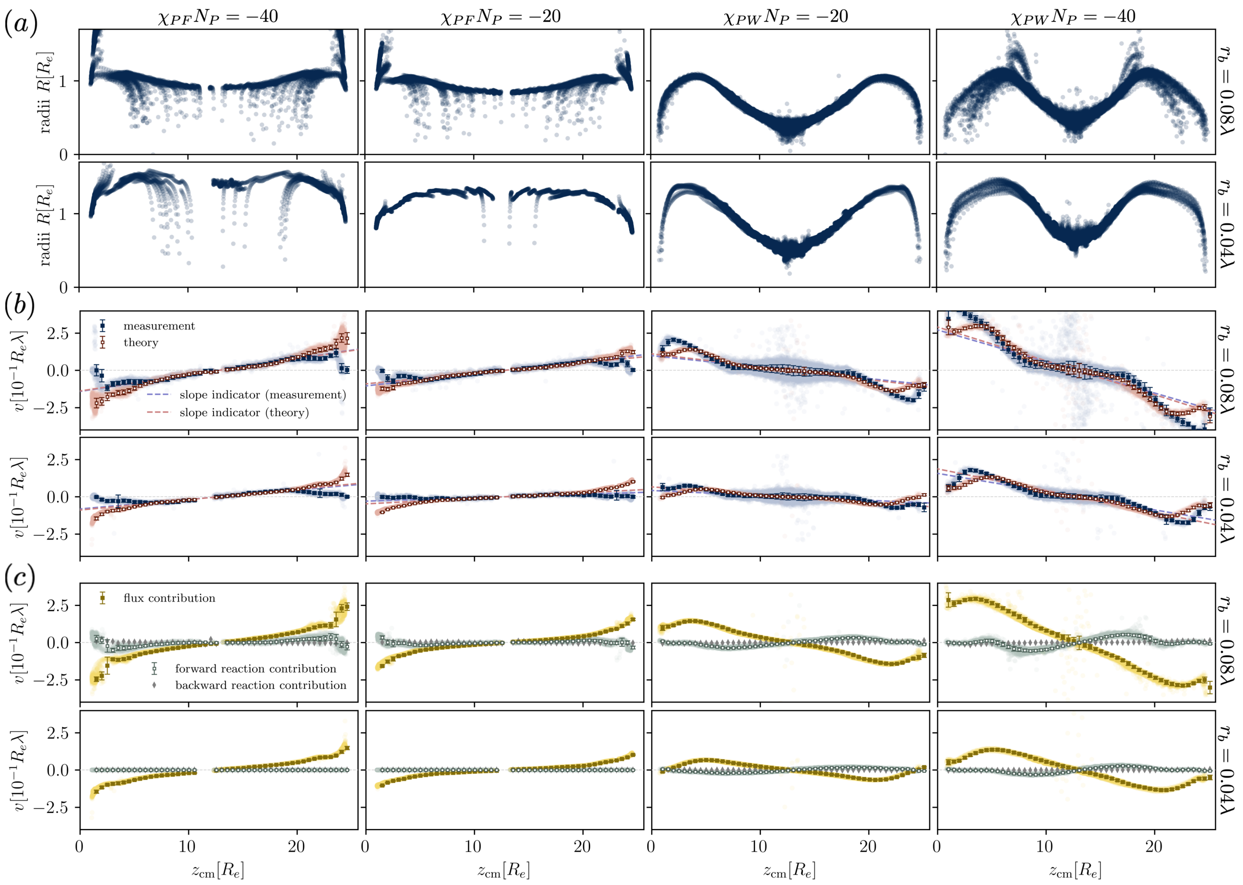

Figure S9: Simulation results for explicit treatment of fuel and waste for multiple system parameters, varying fuel and waste interactions with the product, $\chi_{PF}N_P$ and $\chi_{PW}N_P$, along the columns, and reactions rate constants, r_b, along rows. Among the interactions, one interaction (not indicated in the figure) is set to 0 (nonpreferential). Each quantity is plotted against the droplet center of mass, z_{cm}. (a) Measured droplet radius, R. (b) Measured and theoretically computed droplet velocity, v, using Equation 4. A linear fit in the bulk, $5 \leq z \leq 21.6R_{\mathrm{e}}$, is shown for each curve. (c) Dissection of theoretical velocity into its three contributions. In (b) and (c) the colors and markers are the same as in Figure 5.

panel (b). Clearly strong attractions lead to higher velocities, as well as a higher reaction rate, which is associated with higher concentration gradients. The linear fit in the bulk, $5 \leq z \leq 21.6R_{\mathrm{e}}$, is indicated as dashed lines for both measurement and theory. Its slope is used as the indicator for the droplet dynamics in the main text. Panel (c) dissects the theoretical comparison into the three contributions and we obtain the same results, as in the cases of implicit treatment. The flux contribution determines the overall dynamics of the droplets which becomes stronger, the more attractive one of the component becomes. This determines the arrangement of droplets and how these share the product that is created in the surrounding solution. That way, the forward reaction contribution is enslaved to the flux contribution and acts as a small correction to the velocity. The backward reaction contribution is always negligible.

Particle-based Simulations

To corroborate the continuum model results, we perform particle-based simulations using a soft, coarse-grained model. We use the Monte-Carlo simulation program SOft coarse-grained Monte carlo Acceleration (SOMA)[60] which employs the single-chain-in-mean-field (SCMF) algorithm.[59] The model treats molecules as Gaussian chains, with a discretization N_i, lumping several monomer repeat units into a single interaction center and employs a top-down approach to assess the interactions and dynamics. The model allows for great parallelization by splitting interactions into strong bonded interaction and weak non-bonded ones, $\mathcal{H} = \mathcal{H}_{\mathrm{b}} + \mathcal{H}_{\mathrm{nb}}$ with

$$\frac{\mathcal{H}_{\mathrm{b}}}{k_{\mathrm{B}}T} = \sum_{m} \sum_{b} \frac{3(N_P - 1)}{2R_{\mathrm{e}}^2} (\mathbf{r}_{m,b} - \mathbf{r}_{m,b+1})^2, \tag{S25}$$

where m runs over all molecules, b over all bonds within the molecule, and $\mathbf{r}_{m,b}$, $\mathbf{r}_{m,b+1}$ refer to the positions of the bonded particles. The weak non-bonded interactions are expressed in

terms of the normalized densities $\phi_c(\mathbf{r})$, *i. e.*,

$$\frac{\mathcal{H}_{\rm nb}}{\sqrt{\bar{\mathcal{N}}}k_{\rm B}T} = \int \frac{{\rm d}\mathbf{r}}{R_{\rm e}^3} \left(\frac{\kappa_0 N_P}{2} \left[\sum_c \phi_c(\mathbf{r}) - 1 \right]^2 + \frac{1}{2} \sum_{c \neq c'} \chi_{cc'} N_P \phi_c(\mathbf{r}) \phi_{c'}(\mathbf{r}) \right), \tag{S26}$$

where $\kappa_0 = 6$ is the inverse isothermal compressibility which is large for the nearly incompressible system. A more detailed description of the particle-based model including the implementation of reactions is given in Refs.[52,60]

Conveniently, the two models operate on the same set of parameters which simplifies comparison between the two models. In the particle-based simulations, we take the same parameters, with the exception that solvent, fuel and waste molecules are dimers, $N_{S/F/W} = 2$, just as was done in.[52,61] To comply with this, we also take a higher discretization of the precursor and product molecules, $N_{R/P} = 20$. We take simulation cells of $V = 10R_{\rm e} \times 10R_{\rm e} \times 25R_{\rm e}$ at a resolution of 8 grid cells per $R_{\rm e}$. We take a reaction rate constant of $r_b = 0.08\tau_0^{-1}$ and vary the fuel and waste interactions with the product molecules. Since product and short fuel and waste molecules have different lengths, the invariant interaction parameter is $\chi_{P,F/W}$ rather than $\chi_{P,F/W}N_P$ as was the case if both species had the same discretization. Therefore, we vary the interactions in the range $\chi_{P,F/W} = 0, -2, -4$ to match the interaction of the continuum model with half the discretization values. The results are presented in Figure S10, where we depict the temporally and laterally averaged concentrations in- and outside the droplets in (a) and the measured velocities in (b). Visibly, we obtain the same result as before. The affinity towards one component determines the direction of the movement which can be amplified by high affinities.

Simulations of Multiple Droplet Types

The system presented in Sec. Multiple Droplet Types features two droplet molecule types, P and Q, which phase separate into two distinct droplet phases. Here, we present the

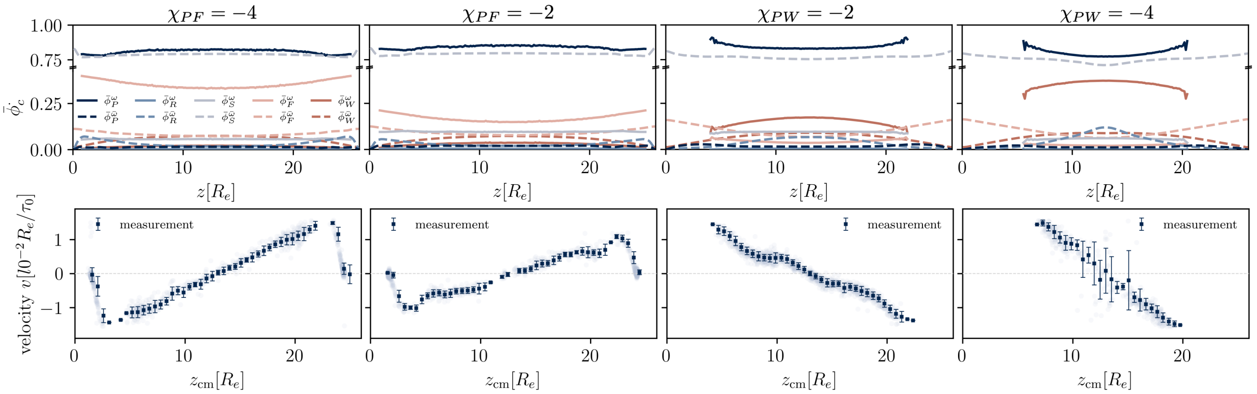

Figure S10: Results of the particle-based simulations for reaction rate constant $r_b = 0.08\tau_0^{-1}$ and variation of interaction parameters. (a) Domain-averaged densities for selected components. (b) Measured individual (light blue circles) and averaged (blue squares) velocities.

parameters that are used and how the simulations are set up. We take a simulation from Sec. RDA-formed Droplets with Explicit Fuel and Waste where the product droplets are attractive towards fuel, $\chi_{PR}N_P = 20, \chi_{PS}N_P = 50, \chi_{PF}N_P = -20, \chi_{PW}N_P = 0$. Using the final state of that simulation as input, we replace a part of the solvent in the system by another homopolymer, Q, such that it makes up 5% of the system's volume. The new component phase separates but favorably interacts with the waste, $\chi_{QR}N_P = 20, \chi_{QS}N_P = 50, \chi_{QF}N_P = 0, \chi_{QW}N_P = -20$. In order to observe two types of droplets that do not wet each other, we take a strong repulsion of P and Q polymers, $\chi_{PQ}N_P = 200$. We show that upon demixing from solution, the Q-rich droplets move towards the center, whereas the P-rich droplets continue to move towards the fuel source. This is demonstrated in Figure 9 of the main text, where example snapshots and velocity measurements demonstrate that even the Q-rich droplets whose components are not involved in the reaction cycle, exhibit a directed movement.

Simulations of Amphiphilic Product Molecules

When introducing one component into the system that is amphiphilic, we need to extend our continuum model to include this. Modeling the amphiphiles with a theory for diblock

copolymers, we employ the free-energy functional proposed by Uneyama and Doi.[64–66] The amphiphilic diblock copolymer consists of two linear blocks with distinct monomer types, called A and B. For each of these blocks, we introduce a density field, as we do for a other single-block components, *i. e.*, R, S, F, W. Introducing for molecular type index p and for the blocks of each molecule index i, the free-energy functional reads

$$\begin{aligned}
\frac{\mathcal{F}\left[\{\phi_{pi}(\mathbf{r})\}\right]}{\sqrt{\bar{\mathcal{N}}}k_{\mathrm{B}}T} &= \frac{1}{R_{\mathrm{e}}^3}\int \mathrm{d}\mathbf{r}\left[\pi(\mathbf{r})N_P\left(\sum_{pi}\phi_{pi}-1\right)\right.\\
&+\sum_{p,ij}\int \mathrm{d}\mathbf{r}'\,2\sqrt{f_{pi}f_{pj}}A_{p,ij}\mathcal{G}(\boldsymbol{r}-\mathbf{r}')\sqrt{\phi_{pi}(\mathbf{r})\phi_{pj}\left(\mathbf{r}'\right)}\\
&+\sum_{pi} f_{pi}C_{p,ii}\phi_{pi}(\mathbf{r})\ln\phi_{pi}(\mathbf{r})\\
&+\sum_{p,i\neq j} 2\sqrt{f_{pi}f_{pj}}C_{p,ij}\sqrt{\phi_{pi}(\mathbf{r})\phi_{pj}(\mathbf{r})}\\
&+\sum_{pi}\frac{R_{\mathrm{e}}^2}{24\phi_{pi}(\mathbf{r})}\left|\nabla\phi_{pi}(\mathbf{r})\right|^2\\
&\left.+\frac{1}{2}\sum_{pi,qj}\chi_{pi,qj}N_P\phi_{pi}(\mathbf{r})\phi_{qj}(\mathbf{r})\right] \qquad \text{(S27)}
\end{aligned}$$

where the coefficients Ap, ij and C_{pij} and the parameters f_{pi} are dependent on the molecular architecture. For simple homopolymers and solvents, the theory reduces to the Flory-Huggins-de Gennes model[41] as introduced in the main text, $A_p = 0$, $C_p = N_P/N_p$ and $f_p = 1$. For the diblock copolymer, the free-energy expansion to second order in the concentrations matches Ohta-Kawasaki free-energy.[67] For these components, the coefficients become[52,61]

$$A_p = \frac{9N_P^2}{N_p^2R_{\mathrm{e}}^2f_{\mathrm{A}}^2f_{\mathrm{B}}^2}\begin{pmatrix} f_{\mathrm{B}}^2 & -f_{\mathrm{A}}f_{\mathrm{B}} \\ -f_{\mathrm{A}}f_{\mathrm{B}} & f_{\mathrm{A}}^2 \end{pmatrix} \qquad \text{(S28)}$$

$$C_p = \frac{N_P}{N_pf_{\mathrm{A}}f_{\mathrm{B}}}\begin{pmatrix} \tilde{s}(f_{\mathrm{A}}) & -\frac{1}{4} \\ -\frac{1}{4} & \tilde{s}(f_{\mathrm{B}}) \end{pmatrix} \qquad \text{(S29)}$$

where $f_{\mathrm{B}} = 1 - f_{\mathrm{A}} = 0.7$ is the block ratio and the definition

$$\tilde{s}(f) = \frac{s(f) - f}{4f(1-f)}. \tag{S30}$$

The reactions are implemented as described in Ref.[52] which assumes a single reaction to convert the hydrophilic precursor into an amphiphilic molecule. This would correspond to short molecules with presumably weak amphiphilicity, where a single reaction can create such a drastic change in the molecule's interactions. Overall, we still describe the reaction cycle by a forward and backward rate constant, r_f and r_b, respectively. The system consists finally of a hydrophilic precursor, R, in solution, S, that reacts with a fuel molecule, F, to become amphiphilic, *i. e.*, the tail, B, becomes hydrophobic, while the head group, A, stays hydrophilic. Waste, W, is produced within the forward reaction and the backward reaction from amphiphile to precursor happens spontaneously, modeled as a first order reaction. The chain discretizations are taken as in the main text, the amphiphilic molecules have a block ratio of $f_A = 1 - f_B = 0.3$, and we take the interaction matrix

$$\chi_{cc'} N_P = \begin{array}{c} \begin{matrix} A & B & R & S & F & W \end{matrix} \\ \begin{pmatrix} 0 & 20 & 0 & -5 & -5 & 20 \\ 20 & 0 & 20 & 50 & 50 & 50 \\ 0 & 20 & 0 & 0 & 0 & 0 \\ -5 & 50 & 0 & 0 & 0 & 0 \\ -5 & 50 & 0 & 0 & 0 & 0 \\ 20 & 50 & 0 & 0 & 0 & 0 \end{pmatrix} \begin{matrix} A \\ B \\ R \\ S \\ F \\ W \end{matrix} \end{array} \tag{S31}$$

We take larger simulation-cell sizes of $V = 51.2R_{\mathrm{e}} \times 9.6R_{\mathrm{e}} \times 9.6R_{\mathrm{e}}$ such that micelles do not grow too quickly after nucleation, but slowly grow upon moving from the bulk towards the source, since the head-groups interact favorably with the fuel. We take a backward reaction

rate constant of $r_b = 0.04\lambda$ and refilling rate constants of $r_{\text{source}} = 0.02\lambda R_\text{e}$, $r_{\text{sink}} = 10\lambda R_\text{e}$. The result is a movement of aggregates as depicted in Sec. Growing complex reaction-driven assemblies, where the slow growth allows the assembly of vesicles, that would not form if it the amphiphiles would just experience a quench into the phase separating parameter regime.

Indeed, performing simulations without fuel and waste and with either no reactions or first-order reactions between precursor and product, but taking all interactions to be the same, we observe no coarsening after the quench and initial nucleation of small micelles, see Figure S11. This also becomes visible when plotting micelle radii against time, where the sizes just stall at the initially unstable length scale and no fusion happens.

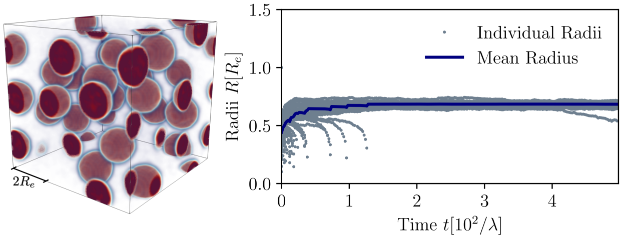

Figure S11: Simple quench test of amphiphiles in equilibrium with the interactions kept the same. Left: Morphology of tail-groups, $\phi_B(\mathbf{r})$ after time $t\lambda = 500$. Right: Time evolution of micelle radii.

Analysis Procedure

The evaluation of the theoretical prediction of Sec. Theoretical Prediction of Velocities requires proper analysis of the simulation data because we use the concentration and flux measurements to compare these to the velocity measurements. Here, we describe step-by-step what is necessary for the simulations and to analyze the (noisy) simulation results.

Simulation As of our implementation of the Uneyama-Doi model (UDM) (available open source *via* `www.gitlab.com/g.ibbeken/udm`), the simulation is a two-step procedure:

1. An Extensible Markup Language (XML) input file is used specify the system architecture (including simulation cell size, grid spacing, external field) the different molecular

components (including molecular volume and their initial mean concentrations), as well as the reaction dynamics. The software provides a script to create a Hierarchical Data Format version 5 (HDF5) file which is the input to the simulation.

2. The HDF5-file is given to the simulations which requires the presence of a compute unified device architecture by Nvidia® (CUDA)-graphics processing unit (GPU). The concentration fields are regularly saved according to an interval, $\Delta\tau$, as specified in the XML input file.

The HDF5 file now contains the simulation results in the form of the time-dependent (n_t saved times) density fields for all n_c components. In the file a $n_t \times n_c \times n_z \times n_y \times n_x$ sized array is saved.

Analysis The analysis takes the time-dependent data and performs the following steps

1. Perform a HKCA on all time-steps, defining a cluster where $\phi_P > 0.5$. The result, a cluster map, is saved in the HDF5-file as a $n_t \times n_z \times n_y \times n_x$-sized array which is zero if no cluster is detected and non-zero, indicating the cluster index, otherwise.

2. Using the HKCA, calculate the cluster radii, R, as described in Ref.[52] and the center of mass, $\mathbf{r}_{\mathrm{cm}}$.

3. Using the center of mass, compute the Voronoi tessellation by determining for each grid cell the cluster with the closest center of mass. The result is stored to the HDF5-file.

4. If it is of interest, the Voronoi-domains can be analyzed in detail. For this the domain is separated into 51 bins (bin width of $\sim 0.5R_{\mathrm{e}}$). Within each bin, we can averaged each Voronoi domain within the center-of-mass frame. From this average, the averaged volume in front of ($z < z_{\mathrm{cm}}$) or behind ($z > z_{\mathrm{cm}}$) the droplet can be computed.

5. Using the HKCA, the concentration fields can be averaged in- and outside of the droplet phases. The cluster analysis separates the droplet from its outside exactly at

the interface. Hence, simply taking averaged concentration where the cluster map is non-zero results in serious miss-evaluation of the inside concentrations of droplets, if these are small (high surface-to-volume ratio). Instead we refine the cluster, making them smaller by excluding grid cells that are (next-nearest-)neighbors of non-cluster grid cells. Averaging over the refined clusters now excludes the interface, leading to clean concentration profiles. For the analysis of the outside concentration profiles the opposite refinement is performed.

6. The velocities are measured using the center-of-mass measurement. For this, the same droplet within two time-frames are detected by finding for each cluster (droplet) a cluster with the closest center of mass (with distance smaller than $|\mathbf{r}_{\mathrm{cm},i,t} - \mathbf{r}_{\mathrm{cm},i',t+\Delta\tau}| \overset{!}{<} R_{\mathrm{e}}$ for cluster i at time t and cluster i' at time $t + \Delta\tau$). The difference in center of mass then determines the velocity.

7. Using Equation 4, the predictions are calculated using the above analysis. To obtain the flux inside the droplets, the noisy data has to be filtered (to obtain clean gradients). To this end, we run the continuum model simulation without thermal noise for a short time, $t\lambda = 0.1$ which is long enough to properly filter the fluctuations and short enough to not alter the concentration fields significantly. The fluxes inside the droplets are measured using the cluster analysis. The reaction contribution uses the (smoothed) local concentration fields in combination with the Voronoi tessellation.

Supplementary Movies

Supplementary Movie I SupplementaryMovieI-ImplicitWaste.mp4:

This movie depicts the time evolution of the local product concentration $\phi_P(\mathbf{r})$ for implicit treatment of waste and neutral interactions of fuel and product, $\chi_{PF}N_P = 0$. One can observe the nucleation of droplets in the center and subsequent movement towards the fuel source.

Supplementary Movie II SUPPLEMENTARYMOVIEII-IMPLICITFUEL.MP4:

This movie depicts the time evolution of the local product concentration $\phi_P(\mathbf{r})$ for implicit treatment of fuel and neutral interactions of waste and product, $\chi_{PW}N_P = 0$. One can observe the nucleation of droplets near the waste sink at the sides of the simulation cell and the subsequent movement towards the center where they fuse. When they reach the center, they are starved of product material and shrink.

Supplementary Movie III SUPPLEMENTARYMOVIEIII-SYMMETRICINTERACTIONS.MP4:

This movie depicts the time evolution of the local product concentration $\phi_P(\mathbf{r})$ for explicit treatment of fuel and waste with nonpreferential interactions of both, waste and product, $\chi_{PF}N_P = \chi_{PW}N_P = 0$. This is the system described in Sec. Fuel and Waste Fluxes Cancel in the Symmetric Case. No net movement can be observed since fuel and waste flux cancel and the reaction contributions are not strong enough to overcome the force asserted by the external potential (wall).

Supplementary Movie IV SUPPLEMENTARYMOVIEIV-ATTRACTIVEFUEL.MP4:

This movie depicts the time evolution of the local product concentration $\phi_P(\mathbf{r})$ for explicit treatment of fuel with favorable interactions between fuel and product, $\chi_{PF}N_P = -40$, and neutral interactions between waste and product, $\chi_{PW}N_P = 0$, at reaction rate constant $r_b = 0.08\lambda$. As described in Sec. RDA-formed Droplets with Explicit Fuel and Waste, for this scenario droplets move towards the fuel source on the side of the simulation cell.

Supplementary Movie V SUPPLEMENTARYMOVIEV-ATTRACTIVEWASTE.MP4:

This movie depicts the time evolution of the local product concentration $\phi_P(\mathbf{r})$ for explicit treatment of fuel with neutral interactions between fuel and product, $\chi_{PF}N_P = 0$, and favorable interactions between waste and product, $\chi_{PW}N_P = -40$, at reaction rate constant $r_b = 0.08\lambda$. As described in Sec. RDA-formed Droplets with Explicit Fuel and Waste, for this scenario droplets move away from the waste sink towards the center of the simulation

cell.

Supplementary Movie VI SupplementaryMovieVI-RDAandPassiveCombined.mp4:

This movie depicts the combined time evolution of the reaction cycle's product concentration $\phi_P(\mathbf{r})$ and the passive macromolecular concentration $\phi_Q(\mathbf{r})$ in the form of the combined order parameter field $\phi_P(\mathbf{r}) - \phi_Q(\mathbf{r})$. The product has an affinity for the fuel, $\chi_{PF}N_P = -40$ and $\chi_{PW}N_P = 0$, while Q has an affinity for waste, $\chi_{PF}N_P = 0$ and $\chi_{PW}N_P = -40$. The two droplet materials strongly repel each other, so effectively no composite phases form, $\chi_{PW}N_P = 200$. As initial condition, we take the final state of Supplementary Movie IV and replace part of the solvent S, such that the Q-molecules make up 5% of the system's volume. As described in Sec. Multiple Droplet Types, while the P-rich droplets continuously nucleate in the center, and move towards the source, the Q-rich, passive droplets quickly nucleate in the whole simulation cell and are transported to the center, where they coalesce and stay.

Supplementary Movie VII SupplementaryMovieVII-AmphiphilicProduct.mp4:

This movie depicts the time evolution of the majority block concentration, $\phi_B(\mathbf{r})$ of Sec. Growing complex reaction-driven assemblies of the main text. All parameters are taken as described in Sec. Simulations of Amphiphilic Product Molecules. One can observe the nucleation in the center, the micelle-to-vesicle transition upon approaching the source and the shrinkage when the vesicles become densely packed at the interface.
